# WHO global research priorities for traditional, complementary, and integrative (TCI) medicine: an international consensus and comparisons with LLMs

**DOI:** 10.7189/jogh.15.04336

**Published:** 2025-11-14

**Authors:** Sangyoung Ahn, Jiali Zhou, Denan Jiang, Steven Kerr, Yajie Zhu, Peige Song, Igor Rudan, WHO TCI medicine CHNRI group, Richard Hammerschlag,, Nicole Skoetz, Shriram Savrikar, Harry HS Fong, Li Shaoping, Pravit Akarasereenont, Holger Cramer, Sabah J Salih, Mei Wang, Caroline Smith, Stefano Masiero, Miek Jong, Elanchezhiyan Devarajan, John Hughes, William Tsang, Lam Fu Chong, Chen Xin, Wang Chun Ming, Zheng Li, Wu Qibiao, Li Ting, Yang Lu, Jennifer AM Stone, Christian Kessler, Immanuel Krankenhaus Berlin, Jeremy Ng, Tammy J Sajdyk, Olobayo Kunle, Suzanne B Hanser, Jacqueline Wiesner, Alicia Catabay, Elaine Elisabetsky, Juergen Barth, Elena Shefer, Goh Cheng Soon, Rokia Sanogo, Sungmin Park, Ikhlas A. Khan, Theodoros Sklaviadis, Claudia Witt, BIAN Zhaoziang, Sanghoon Lee, Charlie Xue, Eleni Skaltsa, Alan Bensoussan, Pierre Duez, Arya Nielsen, Jianping Liu, Jatavallabha Sastry, Gloria Yeh, Maria das Gracas Lins, Martha Villar, Chun-Tao Che, Hui Mo

**Affiliations:** 1 National Institute for Korean Medicine Development, Gyeongsan-si, Republic of Korea; 2 School of Public Health, The Second Affiliated Hospital, Zhejiang University School of Medicine, Hangzhou, Zhejiang, China; 3 International School of Medicine, International Institutes of Medicine, Zhejiang University, Yiwu, Zhejiang, China; 4 Centre for Medical Informatics, Usher Institute, University of Edinburgh, Edinburgh, Scotland, UK; 5 School of Information Science and Technology, Hangzhou Normal University, Hangzhou, Zhejiang, China; 6 Centre for Global Health, Usher Institute, University of Edinburgh, Edinburgh, Scotland, UK; 7 Nuffield Department of Primary Care Health Sciences, Oxford University, Oxford, UK; 8 Green Templeton College, Oxford University, Oxford, UK

## Abstract

**Background:**

Traditional, complementary, and integrative (TCI) medicine is an essential component of health systems worldwide, especially in low- and middle-income countries. Despite its widespread use, existing research on the safety, efficacy, and integration of TCI medicine within conventional healthcare systems is fragmented. This fragmentation highlights the urgent need for a clearly defined global research agenda to guide future studies, secure funding, and inform governance in this field.

**Methods:**

The Traditional, Complementary, and Integrative Medicine Unit at the World Health Organization Headquarters in Geneva, Switzerland coordinated an international research priority-setting exercise using the Child Health and Nutrition Research Initiative (CHNRI) method between June and December 2023. We invited a purposive sample of 120 experts from established academic networks to participate; 53 experts (44.16% response rate) contributed, and 34 of them scored 157 unique research ideas according to five CHNRI criteria: feasibility, effectiveness, deliverability, equity, and potential for disease burden reduction. Additionally, we performed a comparative analysis by generating research priorities using large language models (LLMs), including ChatGPT-4o, Claude 3.5, and Grok 3, and these outputs were compared with the expert-derived priorities.

**Results:**

Top-ranked research priorities focused on chronic disease management (*e.g.* diabetes, dyslipidemia), geriatric safety (*e.g.* herb-drug interactions), mental health (*e.g.* resilience and mood disorders), and integration of TCI into health systems. Priorities varied by income setting. Comparison with LLM-generated lists showed thematic overlap in efficacy and safety but divergence in focus, with LLMs emphasising research capacity, policy, and systems-level priorities.

**Conclusions:**

We established a global, expert-informed research agenda to guide the future direction of TCI medicine and ensure alignment with public health needs. The comparison with LLMs highlights the complementary potential of artificial intelligence in research governance and agenda-setting.

Traditional, complementary, and integrative (TCI) medicine has been a vital component of healthcare systems worldwide, particularly in regions where access to conventional medicine is limited [1–4]. A considerable proportion of the global population relies on TCI medicine as a primary source of healthcare, with practices such as herbal medicine, acupuncture, mind-body therapies, and traditional healing systems deeply embedded in various cultural and medical traditions [5,6]. In recent years, global interest in TCI medicine has increased, driven by multiple factors including the rising burden of non-communicable diseases (NCDs), the growing challenge of antimicrobial resistance, escalating healthcare costs, and persistent gaps in health service coverage, especially in low-resource settings [4,5,7]. As health systems worldwide strive to achieve universal health coverage and address emerging global health challenges, there is a growing need to evaluate how TCI medicine can contribute to holistic, accessible, and cost-effective healthcare solutions [5].

Despite its widespread use, the scientific evidence supporting TCI medicine remains fragmented, and its integration into national health policies varies considerably across countries [2,3]. While some nations have developed regulatory frameworks, institutional research programmes, and standardised clinical guidelines for TCI medicine, others lack systematic approaches for evaluating its efficacy, safety, and economic impact [4,8]. Key unresolved research ideas pertain to the clinical effectiveness of traditional medicine interventions, their mechanisms of action, potential adverse effects, and appropriate integration into existing healthcare systems [1,9]. Furthermore, the absence of globally accepted quality control standards for TCI products and practices raises concerns regarding safety, consistency, and ethical considerations [2]. Addressing these gaps is essential to ensure that TCI medicine is scientifically validated, safely implemented, and appropriately integrated into mainstream healthcare systems.

Although previous research efforts have attempted to address some of these challenges, progress has been hampered by persistent methodological and structural barriers. Systematic reviews and meta-analyses on TCI medicine have often found inconsistencies in study designs, heterogeneity in outcome measures, and a lack of large-scale, high-quality randomised controlled trials [4]. Limited funding for TCI medicine research, coupled with fragmented collaboration between traditional medicine practitioners and biomedical researchers, has further impeded the generation of policy-relevant evidence. Policymakers and health organisations have called for greater methodological standardisation, enhanced data-sharing platforms, and interdisciplinary approaches to bridge the gap between traditional knowledge systems and modern scientific research [2,8]. Without a coherent global research agenda, progress in evidence-based TCI medicine remains slow, limiting its potential contributions to public health and healthcare innovation.

Recognising the need for a coordinated and evidence-informed approach to TCI medicine, the World Health Organization (WHO) has long advocated for strengthening research, policy, and regulation in this field. The WHO Traditional Medicine Strategy (2014–23) emphasised the importance of scientific rigour, innovation, and evidence-based policymaking to optimise the potential benefits of TCI medicine while ensuring safety, efficacy, and equitable access [10]. To advance this agenda, the WHO has facilitated international collaborations, expert consultations, and policy dialogues to advance TCI medicine research. In response to ongoing knowledge gaps, the WHO initiated a global research priority-setting exercise to systematically identify the most critical areas for future research, ensuring alignment with scientific, policy, and health system needs.

Priority-setting exercises, while essential, are often time-consuming and resource-intensive, requiring coordination among multiple stakeholders over extended periods. In this context, recent advances in artificial intelligence (AI), particularly large language models (LLMs), which can rapidly synthesise literature, identify knowledge gaps, and generate candidate research ideas [11–14], offer promising tools to support such processes [15]. While their application in health research is still emerging, early studies suggest that LLMs may offer complementary insights alongside expert-driven approaches in setting research priorities [12,16,17].

We aimed to identify the most pressing research needs and establish research priorities in TCI medicine to guide future research investments and inform policy decisions. Using the Child Health and Nutrition Research Initiative (CHNRI) method, we engaged global experts and stakeholders through a structured prioritisation process. Additionally, we explored the role of AI in research prioritisation by comparing expert-driven research priorities with those generated by LLMs.

## METHODS

### Overview

The TCI Medicine Unit at the WHO Headquarters in Geneva, Switzerland, coordinated this study in collaboration with global experts. We followed the CHNRI method, a widely validated, multi-step framework for research priority setting [18]. Initially developed for child health research, the CHNRI method provides a structured and transparent approach to identifying research priorities and has been employed in over 200 priority-setting exercises across various health fields, including NCDs, infectious diseases, maternal and child health, and health systems research [19]. The CHNRI method is based on crowdsourcing principles, allowing a diverse group of experts to independently propose research ideas and evaluate them using predefined criteria. By incorporating independent assessments from multiple experts, this method minimises individual biases and ensures that research priorities are determined through broad expert consensus rather than individual opinions [18,20]. It has been widely used to inform research agendas for national governments, major funding bodies, and multilateral organisations, including WHO and the United Nations International Children’s Emergency Fund [21,22].

**Figure 1:**
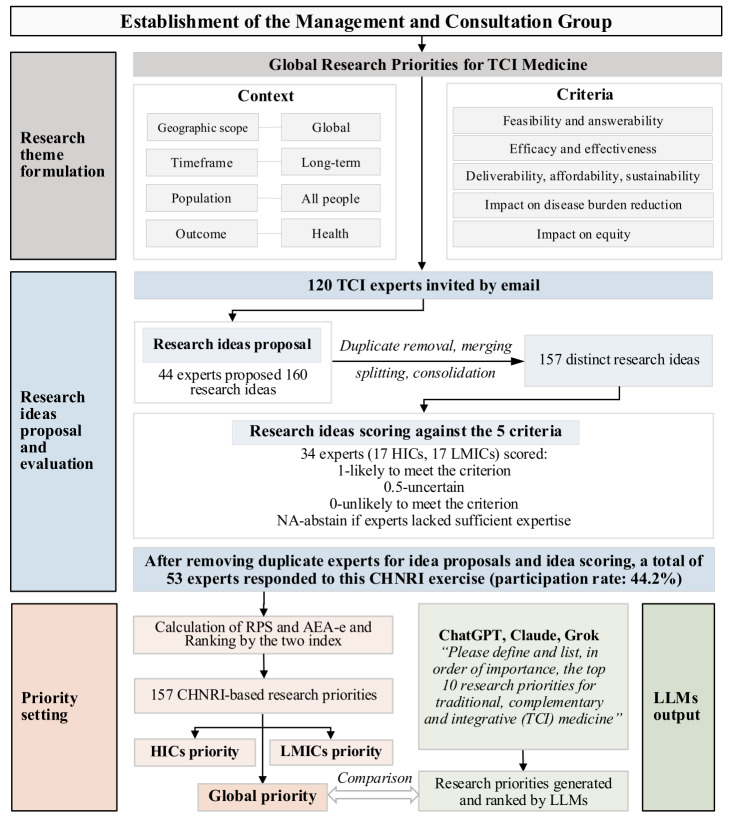
Flowchart of CHNRI exercise. AEA-e – average expert agreement score based on information theory, CHNRI – Child Health and Nutrition Research Initiative, HICs – high-income countries, LLMs – large language models, LMICs – low- and middle-income countries, RPS – research priority score, TCI – traditional, complementary and integrative.

The CHNRI method was applied to TCI medicine, following established methodological approaches to ensure alignment with previous global research prioritisation exercises [19,20,23]. The process involved theme formulation, idea generation, structured scoring, and priority ranking (**Figure 1**).

### Establishment of the management and consultation group

To oversee and coordinate the prioritisation process, we established a management and consultation group (MCG), comprising WHO staff and researchers with expertise in TCI medicine. The MCG was responsible for defining the research theme (*e.g.* objectives, scope, and criteria), coordinating expert engagement across stages, refining and consolidating submitted research ideas for relevance and clarity, developing a structured scoring framework, setting timelines, and facilitating data analysis. The MCG developed a detailed protocol to guide the process.

### Research context and criteria

The MCG defined the research theme as ‘global research priorities for TCI medicine’. Following best practices from previous CHNRI exercises [19], we set the geographic scope as global, ensuring broad applicability of the research priorities across diverse health systems and populations. Recognising TCI medicine’s growing role in addressing challenges such as NCDs, antimicrobial resistance, and universal health coverage gaps, we set a long-term timeframe for anticipated research impact. We defined the target population as the global population, with health improvement as the primary outcome. Following two rounds of MCG discussions, we selected five evaluation criteria, consistent with previous CHNRI studies [24], to assess research ideas:

− Criterion 1: Feasibility and answerability – Can the proposed research be feasibly conducted with current scientific methods and resources?
− Criterion 2: Efficacy and effectiveness – Will the research contribute to generating robust evidence on the efficacy and real-world effectiveness of TCI interventions?
− Criterion 3: Deliverability, affordability, and sustainability – Can the research findings be effectively applied in real-world settings, ensuring that interventions are deliverable, affordable, and sustainable?
− Criterion 4: Maximum impact on disease burden reduction – Will the research significantly contribute to reducing morbidity and mortality?
− Criterion 5: Impact on equity – Will the research improve access to and benefits from TCI medicine for marginalised and underserved populations?

### Invitation of experts

Given TCI medicine’s specialised nature, we purposively selected 120 experts from established networks, including the Cochrane Complementary Medicine Field, the WHO Expert Advisory Panel, the WHO Collaborating Centers, and respondents to an open call advertised globally. We issued the invitations on 30 November 2023, and 44 experts (36.67%) agreed to participate in the first phase. These experts were instructed to submit 3–5 research ideas each, targeting critical knowledge gaps in TCI medicine, such as clinical efficacy, safety, mechanisms of action, health system integration, and policy development.

The 44 participating experts generated 160 research ideas. The MCG then conducted a comprehensive review of these submissions, identifying and removing duplicates, merging conceptually similar topics, splitting overly broad ideas for clarity, and further consolidating overlapping ideas. This process resulted in a final set of 157 unique research ideas.

In the second phase, initiated on 26 May 2024, we circulated this consolidated list of 157 research ideas to the full expert panel (n = 120) for evaluation. Of these, 34 experts (28.33%) submitted completed scores. Experts assessed each idea against the five predefined criteria using a three-point scale: 1 (likely to meet the criterion), 0.5 (uncertain), or 0 (unlikely), with the option to abstain if they lacked sufficient expertise. Scoring instructions emphasised systematic and independent evaluation to minimise bias.

### Data analysis

Before analysis, we anonymised all expert scores and separated them from respondent characteristics to ensure objectivity and protect participant confidentiality, in accordance with ethical research standards. To generate a ranked list of research priorities, we applied equal weighting across all five evaluation criteria. For each research idea, we calculated an intermediate score for each criterion by summing the assigned scores (*i.e.* 1, 0.5, or 0) and dividing by the total number of responses. We excluded unanswered (blank) responses from the denominator to ensure accurate calculations. We then computed the overall research priority score (RPS) for each research idea as the mean of the intermediate scores across the five criteria. This approach ensured a standardised priority ranking, with RPS values theoretically ranging from 0 to 1, where higher scores indicate greater research priority. We calculated RPS as follows:



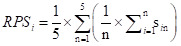



where RPS_i_ is the RPS of the ith research idea.

To quantify the level of agreement among experts, we employed an enhanced average expert agreement score (AEA-e), adapted from previous CHNRI studies and grounded in information theory [25]. Unlike the conventional AEA, which measures consensus based on the proportion of the most frequently assigned score, AEA-e incorporates Shannon entropy to account for the distribution of all assigned scores, providing a more robust measure of consensus [26]. The AEA-e score ranges from 1/3 to 1, where values closer to 1 indicate stronger agreement among experts. We calcultated AEA-e as follows:



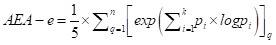



where p_i_ is the proportion of scores in category i (1, 0.5, or 0) for a given research idea, k = 3 represents the number of distinct score categories, n = 5 represents the total number of evaluation criteria, q represents the index of the research idea, ranging from 1 to the total number of research ideas being evaluated.

To quantify uncertainty, we generated 95% confidence intervals (CIs) for both RPS and AEA-e using bootstrap with 1000 iterations. We examined the associations between RPS and AEA-e across the 157 ideas using Spearman’s rank correlation coefficient. To evaluate the consistency between the two-scoring metrics, we also calculated the intraclass correlation coefficient using a two-way mixed-effects model with average measures. Additionally, we performed hierarchical clustering to analyse patterns of scoring diversity, both for overall scores and within individual criteria. To assess potential differences in research prioritisation between experts from high-income countries (HICs) and low- and middle-income countries (LMICs), we conducted subgroup analyses, separately for HIC and LMIC experts. We used Python, version 3.7.1 (Python Software Foundation, Wilmington, Delaware, USA) and *R*, version 4.4.2 (R Core Team, Vienna, Austria) for all analyses.

### Comparison with LLMs

To explore the potential role of AI in research prioritisation, we queried three LLMs – ChatGPT 4o, Claude 3.5 and Grok 3 – on 25 March 2025, with the prompt ‘Please define and list, in order of importance, the top ten research priorities for TCI medicine worldwide’.

### Ethical considerations

This study was approved by the Medical Ethics Committee of the School of Public Health, Zhejiang University (Approval No. ZGL202502-1). We obtained informed consent from all experts through their response to the invitation email and submission of the scoring spreadsheet.

## RESULTS

### Scoring experts demographics

We invited 120 experts to participate in this CHNRI exercise, of whom 53 responded (44.16% participation rate). Among the respondents, 34 participated in scoring (17 from HICs and 17 from LMICs), 25 generated and scoring ideas, nine only scored, and 19 only generated ideas (Figure S1 and Table S2 in the **Online Supplementary Document**).

The overall RPS for the 157 research ideas ranged from 0.408 (95% CI = 0.324–0.489) to 0.903 (95% CI = 0.840–0.944), with a median of 0.649. while the AEA-e varied from 0.365 (95% CI = 0.344–0.392) to 0.727 (95% CI = 0.649–0.807), with a median of 0.474 (Table S3 in the **Online Supplementary Document**). There was strong agreement among experts on the top ten ranked research ideas, with higher RPS rankings corresponding to higher AEA-e rankings (Spearman’s rho = 0.520; *P* < 0.001) (Figure S2 in the **Online Supplementary Document**). No discernible clustering patterns emerged in the overall scoring or across individual criteria (Figures S3–8 in the **Online Supplementary Document**), reflecting the diversity among participating experts and precluding the undue influence of any subgroups.

### Overall top-ranked priorities

The top ten research priorities identified in this study primarily focused on evaluating the efficacy and safety of TCI interventions, particularly in metabolic disorders, mental health, and neurological conditions. More than half of the highly ranked priorities were related to TCI’s role in managing chronic diseases, including diabetes, dyslipidemia, metabolic syndrome, and frailty in the elderly. Mental health research also featured prominently, alongside investigations into acupuncture and manual therapies for neurological and vascular conditions. Additionally, integrating TCI into conventional healthcare systems was recognised as a critical research need. Among these, the top three priorities reflected a strong emphasis on chronic disease management and patient safety. The highest-ranked research priority focused on investigating the effect of traditional medicines on blood sugar control and diabetes (RPS = 0.903; 95% CI = 0.840–0.944; AEA-e = 0.727; 95% CI = 0.649–0.807). The second-ranked priority examined herb-drug interactions in geriatric patients, aiming to minimise adverse effects from commonly used herbal remedies and medications (RPS = 0.876; 95% CI = 0.815–0.919; AEA-e = 0.665; 95% CI = 0.570–0.732). The third priority centred on evaluating the effectiveness of traditional exercise therapies in preventing frailty in the elderly (RPS = 0.846; 95% CI = 0.785–0.897; AEA-e = 0.629; 95% CI = 0.552–0.700). The fourth and fifth priorities investigated TCI applications in dyslipidemia and metabolic syndrome, which were closely related to the top-ranked priority on diabetes management. The sixth and eighth priorities focused on mental health applications of TCI, including psychological resilience and mood disorders. Research on acupuncture and manual therapies for vascular and neurological conditions ranked seventh and ninth, addressing peripheral arterial disease and peripheral neuropathy, respectively. Lastly, the tenth-ranked priority explored barriers and facilitators to integrating TCI approaches into healthcare systems (**Table 1**; Table S3 in the **Online Supplementary Document**).

**Table 1:** The ten highest-ranked research ideas according to RPS and AEA-e

	Research idea	RPS (95% CI)	AEA-e (95% CI)
**Global**			
1	Investigate the effect of traditional medicine on blood sugar control and diabetes	0.903 (0.840–0.944)	0.727 (0.649–0.807)
2	Evaluating the effect of herb-drug interactions on geriatric safety, focusing on commonly used herbal remedies and medications, to minimise adverse effects	0.876 (0.815–0.919)	0.665 (0.570–0.732)
3	Evaluating the effectiveness of the traditional medicine approach to body exercise to prevent frailty in the elderly	0.846 (0.785–0.897)	0.629 (0.552–0.700)
4	Investigate the effect of traditional medicines on dyslipidemias, notably cholesterol and triglyceride levels	0.840 (0.771–0.889)	0.645 (0.589–0.716)
5	Studying the role of integrative traditional medicine (acupuncture, meditation, herbal, lifestyle) approaches in managing metabolic syndrome	0.827 (0.771–0.882)	0.610 (0.542–0.670)
6	Investigating the role of traditional medicine in maintaining or improving mental health and its resilience	0.826 (0.755–0.877)	0.573 (0.501–0.642)
7	Exploring the impact of acupuncture and manual therapies for the treatment of wounds and limb salvage in patients with peripheral arterial disease	0.819 (0.759–0.878)	0.499 (0.435–0.567)
8	Studying the effectiveness of holistic and integrative approaches in treating mood disorders	0.815 (0.752–0.880)	0.599 (0.526–0.654)
9	Exploring the impact of acupuncture, therapeutic massage and movement therapies in seniors with peripheral neuropathy	0.804 (0.740–0.865)	0.535 (0.468–0.602)
10	Identifying barriers and facilitators to integrating traditional medicine approaches into the healthcare system	0.795 (0.727–0.858)	0.555 (0.485–0.619)
**HICs**			
1	Evaluating the effect of herb-drug interactions on geriatric safety, focusing on commonly used herbal remedies and medications, to minimise adverse effects	0.827 (0.737–0.904)	0.596 (0.507–0.699)
2	Investigating the role of traditional medicine in maintaining or improving mental health and its resilience	0.824 (0.729–0.894)	0.628 (0.554–0.713)
3	Investigate the effect of traditional medicines on blood sugar control and diabetes	0.822 (0.712–0.904)	0.626 (0.556–0.708)
4	Evaluating the effectiveness of the traditional medicine approach to body exercise to prevent frailty in the elderly	0.809 (0.715–0.877)	0.581 (0.489–0.661)
5	Identifying barriers and facilitators to integrating traditional medicine approaches into the healthcare system	0.802 (0.714–0.883)	0.554 (0.469–0.644)
6	Studying the effectiveness of holistic and integrative approaches in treating mood disorders	0.800 (0.694–0.871)	0.606 (0.549–0.689)
7	Studying the role of integrative traditional medicine (*e.g.* acupuncture, meditation, herbal, lifestyle) approaches in managing metabolic syndrome	0.780 (0.671–0.854)	0.591 (0.535–0.659)
8	Evaluating the effectiveness of mindfulness activities to improve well-being for the elderly	0.759 (0.659–0.841)	0.545 (0.465–0.613)
9	Investigate the effect of traditional medicines on dyslipidemias, notably cholesterol and triglyceride levels	0.753 (0.644–0.836)	0.572 (0.525–0.654)
10	Exploring the impact of acupuncture and manual therapies for the treatment of wounds and limb salvage in patients with peripheral arterial disease	0.750 (0.647–0.838)	0.456 (0.397–0.554)
**LMICs**			
1	Investigate the effect of traditional medicines on blood sugar control and diabetes	0.986 (0.915–1.000)	0.929 (0.775–1.000)
2	Evaluating the contribution of traditional health practitioners in the fight against diseases (Malaria, Tuberculosis and NCDs)	0.950 (0.883–0.986)	0.778 (0.628–0.928)
3	Evaluating the effect of herb-drug interactions on geriatric safety, focusing on commonly used herbal remedies and medications, to minimise adverse effects	0.930 (0.859–0.972)	0.775 (0.650–0.880)
4	Investigate the effect of traditional medicines on dyslipidemias, notably cholesterol and triglyceride levels	0.930 (0.845–0.972)	0.775 (0.666–0.880)
5	Standardising acupuncture methods in the treatment of stroke	0.926 (0.853–0.971)	0.669 (0.522–0.811)
6	Clinical trials towards evidence-based application of acupuncture in the treatment of chronic pain and a wide range of other conditions	0.903 (0.826–0.951)	0.630 (0.504–0.751)
7*	Evaluating the role of traditional medicine in symptom management for cancer patients, especially in palliative care	0.901 (0.817–0.958)	0.725 (0.621–0.839)
7*	Evaluating the effectiveness of traditional medicine and herbal remedies in improving liver health (preventing the accumulation of fat and cirrhosis)	0.901 (0.817–0.958)	0.725 (0.621–0.839)
9	Exploring the impact of acupuncture, therapeutic massage and movement therapies in seniors with peripheral neuropathy	0.893 (0.821–0.950)	0.601 (0.474–0.707)
10	Evaluating the effectiveness of the traditional medicine approach to body exercise to prevent frailty in the elderly	0.892 (0.800–0.954)	0.711 (0.594–0.829)

AEA-e – average expert agreement score, CI – confidence interval, HIC – high-income country, LMIC – low- and middle-income country, NCD – non-communicable disease, RPS – research priority score

*Tied ranks.

When separated by HICs and LMICs, the top ten research priorities differed (**Table 1**). In HICs, the highest-ranked research priority was evaluating the effect of herb-drug interactions on geriatric safety, focusing on commonly used herbal remedies and medications, to minimise adverse effects (RPS = 0.827; 95% CI = 0.737–0.904; AEA-e = 0.596; 95% CI = 0.507–0.699). The second priority was investigating the role of Traditional Medicine in maintaining or improving mental health and its resilience (RPS = 0.824; 95% CI = 0.729–0.894; AEA-e = 0.628; 95% CI = 0.554–0.713). The globally top-ranked priority, investigating the effect of traditional medicines in blood sugar control and diabetes, ranked third in HICs (RPS = 0.822; 95% CI = 0.712–0.904; AEA-e = 0.626; 95% CI = 0.556–0.708). Other highly ranked topics in HICs included traditional exercise to prevent frailty (4th), barriers and facilitators to TCI integration into healthcare systems (5th), holistic and integrative approaches for mood disorders (6th), integrative TCI approaches for metabolic syndrome (7th), mindfulness activities for elderly well-being (8th), traditional medicines for dyslipidemia (9th), and acupuncture/manual therapy for peripheral arterial disease (10th). In LMICs, the top-ranked priority remained consistent with the global ranking, with research on traditional medicines in blood sugar control and diabetes ranking first (RPS = 0.986; 95% CI = 0.915–1.000; AEA-e = 0.929; 95% CI = 0.775–1.000). The second priority was evaluating the contribution of traditional health practitioners in managing diseases such as malaria, tuberculosis, and NCDs (RPS = 0.950; 95% CI = 0.883–0.986; AEA-e = 0.778; 95% CI = 0.628–0.928). The third-ranked priority in LMICs, evaluating herb-drug interactions in geriatric patients, was ranked first in HICs (RPS = 0.930; 95% CI = 0.859–0.972; AEA-e = 0.775; 95% CI = 0.650–0.880). Other highly ranked topics in LMICs included traditional medicines for dyslipidemia (4th), acupuncture standardisation for stroke (5th), clinical trials on acupuncture for chronic pain (6th), TCI’s role in cancer symptom management and palliative care and herbal remedies for liver health (both ranked 7th), acupuncture and movement therapies for peripheral neuropathy (9th), and traditional exercise-based interventions for frailty prevention (10th).

### Top-ranked priorities across criteria

**Figure 2:**
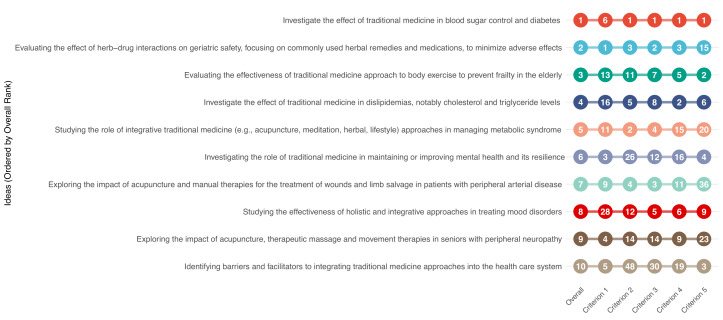
The ranking distribution of the top ten research priorities across all criteria. Criterion 1 – feasibility and answerability, Criterion 2 – efficacy and effectiveness, Criterion 3 – deliverability, affordability, and sustainability, Criterion 4 – maximum impact on disease burden reduction, Criterion 5 – impact on equity.

The top ten research priorities did not always rank consistently across the five evaluation criteria (**Figure 2**). The highest-ranked research priority overall, investigating the effect of traditional medicines on blood sugar control and diabetes, ranked first in efficacy and effectiveness, deliverability, affordability, and sustainability, maximum impact on disease burden reduction, and impact on equity. However, it ranked sixth in feasibility and answerability. The second overall priority, evaluating the effect of herb-drug interactions on geriatric safety, focusing on commonly used herbal remedies and medications to minimise adverse effects, ranked first in feasibility and answerability and also scored highly in deliverability, affordability, and sustainability (2nd), efficacy and effectiveness (3rd), and maximum impact on disease burden reduction (3rd), while it ranked 15th in impact on equity. The third overall priority, evaluating the effectiveness of traditional medicine approaches to body exercise in preventing frailty in the elderly, ranked second in impact on equity, fifth in maximum impact on disease burden reduction, and seventh in deliverability, affordability, and sustainability. However, it ranked outside the top ten in feasibility, answerability, efficacy, and effectiveness.

**Figure 3:**
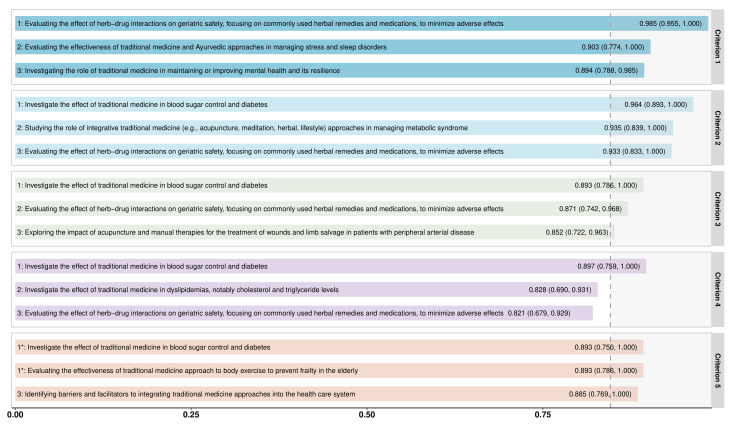
The three highest-ranked research ideas for each of the predefined criteria. Criterion 1 – feasibility and answerability, Criterion 2 – efficacy and effectiveness, Criterion 3 – deliverability, affordability, and sustainability, Criterion 4 – maximum impact on disease burden reduction, Criterion 5 – impact on equity. *Tied ranks. The numbers in the figure represent criterion-specific research priority scores, not the overall score. The dashed vertical line indicates the research priority score cut-off for the 10th overall priority. If a bar extends beyond the dashed vertical line to the right, it means that the research idea ranked within the top ten overall priorities.

For feasibility and answerability, the highest-ranked research priority was evaluating the effect of herb-drug interactions on geriatric safety (**Figure 3**). The second-ranked priority was assessing the effectiveness of traditional medicine and Ayurvedic approaches in managing stress and sleep disorders, which did not appear in the overall top ten. The third-ranked research idea was investigating the role of traditional medicine in maintaining or improving mental health and resilience.

For efficacy and effectiveness, the highest-scoring research priority was investigating the effect of traditional medicines in blood sugar control and diabetes. The second and third-ranked research priorities under this criterion were studying the role of integrative traditional medicine (including acupuncture, meditation, herbal, and lifestyle approaches) in managing metabolic syndrome and evaluating herb-drug interactions in geriatric patients.

For deliverability, affordability, and sustainability, the highest-scoring research priority was investigating the effect of traditional medicines in blood sugar control and diabetes. The second-ranked research priority was evaluating herb-drug interactions in geriatric patients, while the third-ranked priority was exploring the impact of acupuncture and manual therapies on wound treatment and limb salvage in patients with peripheral arterial disease.

To maximise impact on disease burden reduction, the three highest-ranked research priorities were investigating the effects of traditional medicines on blood sugar control and diabetes, evaluating traditional medicines for dyslipidemias (particularly for regulating cholesterol and triglyceride levels), and assessing herb-drug interactions in geriatric patients.

Regarding equity impact, the highest-ranked research priority was investigating the effects of traditional medicines on blood sugar control and diabetes. The second-ranked priority was evaluating the effectiveness of traditional medicine approaches to body exercise in preventing frailty in the elderly. The third-ranked priority was identifying barriers and facilitators to integrating traditional medicine approaches into healthcare systems.

### Comparison with LLMs-generated research priorities

While there were several areas of alignment between CHNRI and the LLM-generated priorities, notable differences also emerged in their focus and emphasis (**Table 2**; Table S4 in the **Online Supplementary Document**). Several overlapping themes were identified. Efficacy and safety evaluation of traditional medicine was a prominent priority across all methods. The CHNRI exercise ranked evaluating herb-drug interactions on geriatric safety as the second-highest priority, while ChatGPT-4o and Claude 3.5 also emphasised efficacy and safety evaluation. Grok 3 included this theme as part of developing standardised research methodologies and protocols, as well as researching the effectiveness and safety of TCI medicine interventions.

**Table 2:** TCI medicine research priorities generated by the three LLMs

Ranking	ChatGPT 4o		Claude 3.5	Grok 3
1	Evidence Generation for Safety and Efficacy	Efficacy and Safety Evaluation		Developing Standardised Research Methodologies and Protocols for TCI Medicine
2	Integration of TCI into Primary Health Care Systems	Mechanistic Understanding		Improving Research Capacity and Training for TCI Medicine Practitioners and Researchers
3	Regulation, Quality Assurance, and Standardisation	Global Health Integration		Advocating for Increased Funding and Resource Allocation for TCI Medicine Research
4	Mechanisms of Action and Translational Research	Patient-Centred Outcomes Research		Conducting Research on the Effectiveness and Safety of TCI Medicine Interventions
5	Addressing NCDs	Pharmacological and Botanical Research		Researching the Efficacy of TCI Medicine for Specific Health Conditions
6	TCI in Mental Health and Well-being	Precision and Personalised Medicine		Researching Policy Development and Implementation for the Integration of TCI Medicine into National Health Systems
7	Indigenous Knowledge Systems and Biodiversity Conservation	Mental Health and Psychological Interventions		Researching Patient-Centred Care and Individualised Approaches in TCI Medicine
8	Personalised and Preventive Health Approaches	Chronic Disease Management		Exploring the Potential of TCI Medicine in Preventive Care and Health Promotion
9	Digital Health and Innovation in TCI	Global Traditional Knowledge Preservation		Investigating the Role of TCI Medicine in Addressing Health Disparities and Improving Access to Care
10	Health Equity, Access, and Cultural Acceptability	Economic and Health System Impact		Researching the Integration of TCI Medicine with Conventional Medicine to Create a More Holistic and Comprehensive Healthcare System

LLM – large language model, NCD – non-communicable disease, TCI – traditional, complementary, and integrative

Traditional medicine for chronic disease management was another shared focus. The top-ranked CHNRI priority, investigating the effect of traditional medicines in blood sugar control and diabetes, was also reflected in ChatGPT-4o’s and Claude 3.5’s outputs, both of which included chronic disease management and metabolic health as key areas. Grok 3 addressed this theme through researching the efficacy of TCI medicine for specific health conditions. Similarly, CHNRI’s priority on dyslipidemia treatment aligned with broader references to chronic disease and metabolic health research in the LLM-generated lists.

Mental health and psychological interventions were widely represented. The CHNRI method ranked investigating the role of Traditional Medicine in mental health and resilience, and studying holistic and integrative approaches for mood disorders, within the top ten. ChatGPT-4o and Claude 3.5 also explicitly listed mental health and psychological interventions, while Grok 3 included related themes under patient-centred care and individualised approaches.

Integration of traditional medicine into healthcare systems was another area of alignment. The CHNRI method identified barriers and facilitators to integrating traditional medicine into healthcare as a key research priority. ChatGPT-4o and Grok 3 both emphasised integration of TCI medicine into primary healthcare systems, while Claude 3.5 included global health integration and economic and health system impact as related themes.

Several differences were also noted. The CHNRI method included evaluating the effectiveness of traditional medicine approaches to body exercise to prevent frailty in the elderly, a priority that did not appear explicitly in the LLM-generated lists. Similarly, research on acupuncture, manual therapies, and movement therapies for specific conditions (*e.g.* wound healing, peripheral neuropathy) was more prominent in CHNRI, whereas the LLMs did not specify these interventions. Conversely, Grok 3 uniquely emphasised research capacity building, funding advocacy, and policy development for TCI medicine, priorities that did not emerge in CHNRI’s top ten.

## DISCUSSION

We identified key research priorities in TCI medicine, with a strong focus on chronic disease management, patient safety, mental health, and healthcare system integration. Among the top-ranked priorities, more than half addressed TCI’s role in managing metabolic disorders, including diabetes (1st), dyslipidemia (4th), and metabolic syndrome (5th), as well as frailty prevention in the elderly (3rd). Herb-drug interactions in geriatric patients (2nd) and mental health applications of TCI (6th and 8th) were also highly prioritised. Research on acupuncture and manual therapies for neurological and vascular conditions (7th and 9th) and barriers to TCI integration into healthcare systems (10th) further highlighted critical gaps. While HICs prioritised geriatric safety and mental health, LMICs emphasised diabetes management, the role of traditional health practitioners, and the standardisation of acupuncture. A comparison with ChatGPT-4o, Claude 3.5, and Grok 3 revealed substantial alignment in efficacy and safety evaluation, chronic disease management, and mental health, but LLMs placed greater emphasis on research capacity building, policy development, and funding advocacy, whereas the CHNRI approach prioritised specific interventions such as acupuncture and movement-based therapies.

The prioritisation of research on traditional medicines for diabetes management (1st) and herb-drug interactions in geriatric patients (2nd) aligns with longstanding research on the global rise of metabolic disorders and the increasing complexity of medication use in ageing populations [25,26]. Diabetes, dyslipidemia (4th), and metabolic syndrome (5th) are leading contributors to the burden of NCDs, and traditional medicine has been widely explored for its potential role in managing these conditions. However, while some herbal and integrative therapies have shown promise in glycaemic control and lipid regulation, concerns regarding safety, standardisation, and herb-drug interactions persist, explaining why both efficacy and safety considerations ranked among the highest priorities [27–29]. Similarly, the third-ranked priority, evaluating traditional exercise therapies for frailty prevention in the elderly, reflects growing evidence supporting movement-based interventions as a key strategy for healthy ageing [30,31]. Research on acupuncture and manual therapies for vascular and neurological conditions (7th and 9th) further underscores the need to assess alternative and complementary treatments for chronic pain, circulation issues, and neuropathic disorders, areas where conventional medicine often has limited long-term solutions [32,33]. Mental health research also featured prominently, with TCI approaches for psychological resilience (6th) and mood disorders (8th) ranking highly, reinforcing a growing global shift toward holistic and integrative mental healthcare [34,35]. Lastly, identifying barriers and facilitators to TCI integration into healthcare systems (10th) highlights ongoing structural and policy challenges, aligning with previous studies emphasising the need for regulatory frameworks, standardisation, and evidence-based guidelines to support the responsible integration of TCI into conventional healthcare settings [2,36,37].

While these rankings suggest a broad global consensus on key research priorities, differences between HICs and LMICs highlight regional variations in healthcare needs and system capacities. In HICs, the highest-ranked priority focused on herb-drug interactions in geriatric patients, reflecting concerns about polypharmacy and patient safety in ageing populations with high medication use. In contrast, LMICs prioritised research on traditional medicine for diabetes, likely due to the high prevalence of diabetes and the need for accessible, cost-effective treatment options [25,26]. Additionally, LMICs placed greater emphasis on the role of traditional health practitioners in disease management and acupuncture standardisation, whereas HICs focused more on mental health applications and healthcare system integration, reflecting differences in healthcare infrastructure, regulatory frameworks, and disease burden.

The distribution of rankings across CHNRI evaluation criteria provides further insight into expert perceptions of feasibility, impact, and implementation potential. The top-ranked research priority, investigating traditional medicines for diabetes, ranked highest across impact-based criteria (*e.g.* efficacy, affordability, and disease burden reduction) but ranked lower in feasibility, likely due to challenges in standardisation, clinical validation, and regulatory approval [3,6]. Conversely, the second-ranked priority, evaluating herb-drug interactions in geriatric patients, ranked highest in feasibility and answerability, suggesting that experts viewed this as an immediately actionable research area with clearer study pathways. The third-ranked priority, traditional exercise therapies for frailty prevention, ranked well on impact on equity, reinforcing its potential to benefit diverse populations; however, its lower feasibility ranking suggests implementation challenges related to adherence, accessibility, and standardisation [38]. These variations across criteria highlight the importance of assessing multiple dimensions of value. In the CHNRI framework, all criteria are treated as important, but their relative weights can be adjusted based on stakeholder input [19,39]. For example, if stakeholders prioritise equity, then research options scoring highly on that criterion can be given greater emphasis in final decision-making [40].

The comparison between the CHNRI-derived research priorities and those generated by ChatGPT-4o, Claude 3.5, and Grok 3 revealed notable similarities, providing reassurance about key research directions, but also significant differences in focus and specificity. Both methods highlighted efficacy and safety evaluation, chronic disease management, mental health, and healthcare system integration as major priorities, demonstrating alignment in recognising critical areas for TCI research. However, CHNRI’s expert-driven process produced more specific and actionable research priorities, such as evaluating traditional exercise therapies for frailty prevention and acupuncture for vascular and neurological conditions, which were less prominent in the LLM-generated outputs. In contrast, LLMs proposed broader and cross-cutting themes, such as digital health, personalised medicine, and global knowledge preservation, and Grok 3 uniquely emphasised research capacity building and policy development, which did not emerge in CHNRI’s top ten. These differences suggest that LLMs may be useful in identifying overarching research landscapes or systemic gaps, while expert input is essential to refine and contextualise priorities based on feasibility and real-world clinical relevance. This complementarity highlights the potential value of integrating both approaches. LLMs may assist horizon scanning and idea generation, while structured expert engagement ensures practical relevance and implementation potential [12,13,17,19,40].

A major strength of this study is the application of the CHNRI method, which provides a transparent, systematic, and replicable approach to identifying and ranking research priorities [19]. This represents the first global consensus effort to establish a clear research agenda for TCI medicine, offering a structured foundation for future studies. By analysing differences in priorities between HICs and LMICs, our findings can inform context-specific policy and funding strategies. Notably, there was strong expert agreement on the highest-ranked research ideas, ensuring the stability and reproducibility of the final rankings [33,37]. No significant clustering patterns emerged in scoring across different criteria, suggesting a broad and diverse range of perspectives. Our response rate is comparable to those reported in similar CHNRI-based priority-setting exercises, and the resulting global participation of experts further strengthened the robustness of our findings [19,23,41]. Additionally, by comparing expert-driven research priorities with those generated by multiple LLMs (ChatGPT-4o, Claude 3.5, and Grok 3), we provide an additional layer of validation and insight into the alignment between AI-generated and expert-identified research needs.

However, this study has certain limitations. The expert panel was composed exclusively of TCI medicine specialists, which ensured topic-specific expertise but may have introduced bias by limiting diversity of perspectives. Future exercises could include a broader range of stakeholders to enhance inclusivity and reduce potential bias. Additionally, the number of scoring experts was 34, slightly below the CHNRI-recommended threshold of 45, which may have affected the rankings’ stability [24]. The HIC and LMIC subgroups each had 17 experts, potentially limiting the robustness of results when comparing regional priorities. Furthermore, the use of a ‘0.5’ response option to indicate uncertainty may have contributed to a regression-to-the-mean effect, potentially reducing differentiation between priorities. While the comparison with LLMs provided useful insights, AI-generated outputs remain in an early stage of development, and their ability to capture emerging research needs, contextual feasibility, and recent clinical challenges remain uncertain. Future studies with larger, more regionally diverse expert panels, along with periodic updates, will be essential for regional findings and comparisons.

Our findings provide a structured foundation for shaping the future research agenda in TCI medicine, offering guidance to researchers, funding bodies, and policymakers. As TCI medicine continues to evolve, targeted investments in high-priority research areas will be essential to generate the evidence base needed for clinical practice, policy formulation, and integration into mainstream healthcare systems. Funding agencies should strategically allocate resources to support studies that address key knowledge gaps, ensuring that research aligns with public health needs and has a real-world impact. This is particularly relevant for global actors such as the WHO, whose Traditional Medicine Strategy and Global Centre for Traditional Medicine can serve as platforms for coordinating funding, aligning research priorities with global health goals, and supporting cross-country collaboration. Given the differences in research priorities between HICs and LMICs, operationalising this agenda will require tailored approaches across different contexts. Funding strategies should be context-sensitive, supporting both globally relevant research and region-specific studies that address local healthcare challenges, especially in LMICs. The WHO regional offices and national health ministries can play an instrumental role by embedding these priorities into national research strategies and aligning them with existing funding streams. Investment in research infrastructure, regulatory frameworks, and capacity-building (*e.g.* training programmes and research networks) will also be essential to enable high-quality TCI research.

From a policy perspective, these research priorities can serve as a roadmap for governments, regulatory bodies, and healthcare institutions to guide decision-making on the integration, standardisation, and regulation of TCI medicines. Policymakers should leverage these findings to develop evidence-informed policies that ensure safe, effective, and sustainable incorporation of TCI medicine into health systems. Moreover, regulatory frameworks should be strengthened to support high-quality research, facilitate ethical clinical trials, and establish standardised guidelines for TCI practice [3]. To maximise the impact of this priority-setting exercise, stronger collaborations among researchers, funders, and policymakers are needed. Establishing global and regional networks for TCI medicine research, under the coordination of the WHO or similar bodies, can facilitate knowledge exchange, data sharing, and harmonised research efforts. Additionally, engaging patients, practitioners, and traditional medicine stakeholders in the research process can enhance acceptability, relevance, and successful implementation of findings into healthcare systems. By translating these priorities into well-funded, high-quality research and evidence-based policies, our findings can advance TCI medicine, bridge traditional knowledge with modern scientific approaches, and ultimately improve healthcare outcomes globally.

## CONCLUSIONS

We systematically identified global research priorities for TCI medicine, highlighting key areas such as chronic disease management, geriatric safety, mental health, and healthcare system integration. The findings reflect both shared global concerns and regional differences between HICs and LMICs, underscoring the need for targeted, context-specific research. While expert-driven prioritisation aligned with AI-generated research themes, the CHNRI approach provided more specific and actionable directions. Addressing these priorities through well-funded, high-quality research and policy initiatives will be essential for advancing evidence-based TCI medicine. As the field continues to evolve, interdisciplinary collaboration and sustained investment will be crucial to ensuring its safe, effective, and equitable integration into global healthcare systems.

## Additional material


Online Supplementary Document
