# Supplementary material for: WHO global research priorities for traditional, complementary, and integrative (TCI) medicine: an international consensus and comparisons with LLMs
Source: J Glob Health. 2025 Nov 14;15:04336. doi: 10.7189/jogh.15.04336 (PMC12615007; doi:10.7189/jogh.15.04336)
Supplement: Online Supplementary Document [file jogh-15-04336-s001.pdf]

Supplement to: Ahn S, Zhou J, Jiang D, Kerr S, Zhu Y, Song P, Rudan I. WHO global research priorities for traditional, complementary, and integrative (TCI) medicine: an international consensus and comparisons with LLMs. *J Glob Health*. 2025;15:04336.

Table S1. Author groups list: WHO TCI medicine CHNRI group 2
Table S2. Summary characteristics of experts contributing to the scoring process in this CHNRI exercise (N=34) 4
Table S3. Full list of the 157 research priorities ranked by their overall RPS 5
Table S4. The output from the three LLMs for TCI medicine research priorities 16
Figure S1. The geographic distribution of contributing experts (N = 53) 25
Figure S2. Correlation between RPS and AEA-e 26
Figure S3. The hierarchical clustering of all idea scores from contributing experts 27
Figure S4. The clustering of idea scores in the Criterion 1 – Feasibility and Answerability 28
Figure S5. The clustering of idea scores in the Criterion 2 – Efficacy and Effectiveness 29
Figure S6. The clustering of idea scores in the Criterion 3 – Deliverability, Affordability, and Sustainability 30
Figure S7. The clustering of idea scores in the Criterion 4 – Maximum Impact on Disease Burden Reduction 31
Figure S8. The clustering of idea scores in the Criterion 5 – Impact on Equity 32

This supplementary material has been provided by the authors to give readers additional information about their work.

Table S1. Author groups list: WHO TCI medicine CHNRI group

Name	Affiliation
Richard Hammerschlag	Nova Institute
Nicole Skoetz	University Hospital Cologne
Shriram Savrikar	Pharmacopeia Commission of India
Harry HS Fong	University of Illinois at Chicago
Li Shaoping	University of Macau
Pravit Akarasereenont	Mahidol University
Holger Cramer	University of Tübingen
Sabah J Salih	University of Al-Ameed
Mei Wang	Leiden University
Caroline Smith	Western Sydney University
Stefano Masiero	University of Padova
Miek Jong	The Arctic University of Norway
Elanchezhiyan Devarajan	Morarji Desai National Institute of Yoga
John Hughes	University College London Hospitals
William Tsang	Hong Kong Metropolitan University
Lam Fu Chong	Pharmaceutical Administration Bureau of Macau
Chen Xin	University of Macau
Wang Chun Ming	University of Macau
Zheng Li	Macau University of Science and Technology
Wu Qibiao	Macau University of Science and Technology
Li Ting	Macau University of Science and Technology
Yang Lu	Traditional Chinese Medicine, University Hospital, Macau
Jennifer AM Stone	Indiana University
Christian Kessler	Immanuel Krankenhaus Berlin
Jeremy Ng	University of Tübingen
Tammy J Sajdyk	Indiana University
Olobayo Kunle	National Institute for Pharmaceutical Research and Development, Abuja Nigeria
Suzanne B Hanser	Berklee College of Music
Jacqueline Wiesner	Federal Institute for Drugs and Medical Devices
Alicia Catabay	De La Salle Medical and Health Sciences Institute
Elaine Elisabetsky	Universidade Federal do Rio Grande do Sul
Juergen Barth	University Hospital Zurich

Elena Shefer	Ministry of Health of the Russian Federation
Goh Cheng Soon	Ministry of Health Malaysia
Rokia Sanogo	University of Sciences, Techniques and Technologies of Bamako
Sungmin Park	National Institute of Food and Drug Safety Evaluation
Ikhlas A. Khan	National Center for Natural Products Research
Theodoros Sklaviadis	Aristotle University of Thessaloniki
Claudia Witt	University Hospital Zurich
BIAN Zhaoziang	Hong Kong Baptist University
Sanghoon Lee	Kyung Hee University
Charlie Xue	RMIT University
Eleni Skaltsa	National and Kapodistrian University of Athens
Alan Bensoussan	Western Sydney University
Pierre Duez	University of Mons
Arya Nielsen	Icahn School of Medicine at Mount Sinai
Jianping Liu	Beijing University of Chinese Medicine
Jatavallabha Sastry	National Medicinal Plant Board
Gloria Yeh	Beth Israel Lahey Health
Maria das Gracas Lins [illegible]	Federal University of Minas Gerais
Martha Villar	Social Health Insurance of Peru
Chun-Tao Che	University of Illinois Chicago
Hui Mo	Health Bureau

Note: *WHO, World Health Organization; CHNRI, Child Health and Nutrition Research Initiative; TCI, traditional, complementary and integrative.*

Table S2. Summary characteristics of experts contributing to the scoring process in this CHNRI exercise (N=34)

Characteristic	Number	Percentage
**Sex**		
Male	19	55.88
Female	15	44.12
**WHO regions**		
African Region	1	2.94
Eastern Mediterranean Region	1	2.94
European Region	10	29.41
Region of the Americas	7	20.59
South-East Asia Region	3	8.82
Western Pacific Region	12	35.29
**World Bank Income Groups**		
HICs	17	50.00
LMICs	17	50.00
**Highest Level of Education**		
Doctor degree	34	100.00

Note: *The 34 experts here were responsible for scoring process (either scoring alone or both proposing and scoring). CHNRI, Child Health and Nutrition Research Initiative; WHO, World Health Organization; HICs, high-income countries; LMICs, low- and middle-income countries.*

Table S3. Full list of the 157 research priorities ranked by their overall RPS

Rank	Research idea	Overall RPS (95%CI)	Overall AEA-e (95%CI)
1	Investigate the effect of traditional medicine in blood sugar control and diabetes	0.903 (0.840, 0.944)	0.727 (0.649, 0.807)
2	Evaluating the effect of herb-drug interactions on geriatric safety, focusing on commonly used herbal remedies and medications, to minimize adverse effects	0.876 (0.815, 0.919)	0.665 (0.570, 0.732)
3	Evaluating the effectiveness of traditional medicine approach to body exercise to prevent frailty in the elderly	0.846 (0.785, 0.897)	0.629 (0.552, 0.700)
4	Investigate the effect of traditional medicine in dyslipidemias, notably cholesterol and triglyceride levels	0.840 (0.771, 0.889)	0.645 (0.589, 0.716)
5	Studying the role of integrative traditional medicine (e.g., acupuncture, meditation, herbal, lifestyle) approaches in managing metabolic syndrome	0.827 (0.771, 0.882)	0.610 (0.542, 0.670)
6	Investigating the role of traditional medicine in maintaining or improving mental health and its resilience	0.826 (0.755, 0.877)	0.573 (0.501, 0.642)
7	Exploring the impact of acupuncture and manual therapies for the treatment of wounds and limb salvage in patients with peripheral arterial disease	0.819 (0.759, 0.878)	0.499 (0.435, 0.567)
8	Studying the effectiveness of holistic and integrative approaches in treating mood disorders	0.815 (0.752, 0.880)	0.599 (0.526, 0.654)
9	Exploring the impact of acupuncture, therapeutic massage and movement therapies in seniors with peripheral neuropathy	0.804 (0.740, 0.865)	0.535 (0.468, 0.602)
10	Identifying barriers and facilitators to integrating traditional medicine approaches into the health care system	0.795 (0.727, 0.858)	0.555 (0.485, 0.619)
11	Evaluating the effect of acupuncture on cardiovascular system in ageing individuals (e.g., impact on blood pressure, circulation, and overall cardiovascular health)	0.792 (0.723, 0.850)	0.488 (0.426, 0.554)
12	Evaluating the effectiveness of mindfulness activities to improve well-being for the elderly	0.788 (0.719, 0.848)	0.539 (0.467, 0.602)
13	Evaluating the role of traditional medicine in symptom management for cancer patients, especially palliative care	0.785 (0.715, 0.843)	0.575 (0.510, 0.631)
14	Clinical trials towards evidence-based application of acupuncture in treatment of chronic pain and a wide range of other conditions	0.779 (0.710, 0.841)	0.533 (0.466, 0.594)

Rank	Research idea	Overall RPS (95%CI)	Overall AEA-e (95%CI)
15	Reviewing existing evidence, conducting new clinical trials, and developing clinical practice guidelines for Chronic Pain management with traditional medicine, from acupuncture to herbs	0.772 (0.698, 0.832)	0.474 (0.420, 0.538)
16	Studying the role of traditional medicine in prevention and treatment of degenerative diseases (e.g., Parkinson's, Alzheimer's, Rheumatoid Arthritis)	0.763 (0.697, 0.827)	0.534 (0.477, 0.590)
17	Evaluating the effectiveness of herbal products for treatment of mild skin disorders	0.763 (0.686, 0.824)	0.502 (0.446, 0.559)
18	Evaluating the use of traditional and complementary therapies in reducing antimicrobial resistance	0.756 (0.683, 0.817)	0.542 (0.486, 0.595)
19	Assessing the effectiveness of TCI-based applications for the self-management of chronic conditions	0.754 (0.677, 0.819)	0.494 (0.433, 0.556)
20	Studying the prevalence and effects of the concurrent use of traditional and synthetic medicines in chronic diseases (e.g., hypertension and diabetes)	0.748 (0.670, 0.810)	0.506 (0.450, 0.567)
21	Evaluating the effectiveness of traditional medicine and Ayurvedic approaches in managing stress and sleep disorders	0.748 (0.674, 0.816)	0.549 (0.494, 0.596)
22	Evaluating the effectiveness of acupuncture for reducing post-traumatic stress disorder in refugees	0.746 (0.669, 0.813)	0.494 (0.444, 0.552)
23	Evaluating the effectiveness of herbal products for mild gastrointestinal ailments	0.746 (0.679, 0.811)	0.455 (0.405, 0.512)
24	Evaluating the contribution of traditional health practitioners in the fight against diseases (Malaria, Tuberculosis and NCDs)	0.745 (0.675, 0.808)	0.488 (0.439, 0.544)
25	Evaluating strategies to promote pragmatic trial designs and pragmatic settings or traditional medicine to make it generalizable and clinically relevant	0.736 (0.652, 0.804)	0.541 (0.485, 0.591)
26	Determining a standardized approach of acupuncture treatment in treating anxiety and depression	0.733 (0.666, 0.797)	0.463 (0.415, 0.514)
27	Evaluating the effectiveness of traditional medicine and herbal remedies in improving liver health (preventing accumulation of fat and cirrhosis)	0.731 (0.652, 0.800)	0.504 (0.452, 0.555)
28	Studying herbal preparations and biological drugs with proven effectiveness for anti-ageing treatment (e.g., memory, attention, bones and joints, cardiovascular system).	0.726 (0.647, 0.794)	0.501 (0.447, 0.566)
29	Evaluating the effect of acupuncture on cognitive function in ageing and exploring its potential in preventing or slowing down cognitive decline and neurodegeneration	0.723 (0.649, 0.788)	0.455 (0.405, 0.505)
30	Evaluating the role of traditional medicine in stroke rehabilitation	0.722 (0.649, 0.788)	0.521 (0.466, 0.570)

Rank	Research idea	Overall RPS (95%CI)	Overall AEA-e (95%CI)
31	Standardising the guidelines for quality of herbal medicine products to facilitate broad and consistent use	0.721 (0.643, 0.786)	0.499 (0.444, 0.551)
32	Conducting multidisciplinary and longitudinal studies of music therapy interventions on clinical outcomes (e.g., anxiety, pain, depression, cognition, quality of life)	0.719 (0.633, 0.793)	0.552 (0.517, 0.601)
33	Developing methodology for clinical research that will enhance translation of outcomes into practice (e.g., definitions, criteria, diagnosis and outcome measures)	0.715 (0.623, 0.782)	0.530 (0.479, 0.572)
34	Studying effectiveness and cost-effectiveness of virtual, online, m-Health/e-Health delivery of complementary and integrative, traditional therapies	0.713 (0.632, 0.774)	0.487 (0.431, 0.540)
35	Identifying factors that prevent the mainstreaming of traditional medicine in health systems in developing countries in spite of their cultural acceptance and popularity	0.712 (0.635, 0.783)	0.464 (0.413, 0.516)
36	Assessing safety and effectiveness of cannabinoids for the self-management of chronic conditions	0.709 (0.627, 0.770)	0.475 (0.426, 0.522)
37	Identifying most appropriate and effective mechanisms to monitor and regulate the quality of herbal products	0.707 (0.627, 0.772)	0.481 (0.431, 0.533)
38	Evaluating the role of Ayurvedic therapeutic interventions in management of dementia and Alzheimer's disease	0.705 (0.624, 0.779)	0.454 (0.402, 0.503)
39	Clinical trials towards evidence-based application of meditation in treatment of chronic pain and a wide range of other conditions	0.705 (0.633, 0.782)	0.490 (0.440, 0.539)
40	Evaluating approaches for conservation and protection of ethnomedicine knowledge and practice	0.705 (0.629, 0.773)	0.487 (0.436, 0.538)
41	Developing internationally accepted standardized guidelines for clinical trials in traditional medicine	0.704 (0.629, 0.776)	0.526 (0.476, 0.565)
42	Evaluating the effectiveness of mindfulness-based therapies for attention deficit and hyperactivity disorder	0.701 (0.620, 0.777)	0.509 (0.457, 0.555)
43	Conducting a comprehensive review and meta-analysis of methodologies in traditional medicine research to enhance its rigor and validity	0.697 (0.616, 0.762)	0.479 (0.434, 0.526)
44	Validating protocols for quality assurance of all herbal source materials in clinical trials	0.697 (0.616, 0.768)	0.487 (0.441, 0.533)
45	Developing educational standards, including knowledge, skills, attributes and literacy, in evidence-based health care and traditional medicine interventions	0.695 (0.624, 0.771)	0.474 (0.423, 0.520)

Rank	Research idea	Overall RPS (95%CI)	Overall AEA-e (95%CI)
46	Studying the efficacy of Yoga and its therapeutic effects in improving quality of life	0.695 (0.620, 0.759)	0.408 (0.374, 0.452)
47	Evaluating the effectiveness of traditional medicines used in the treatment of sickle cell disease	0.695 (0.611, 0.766)	0.541 (0.512, 0.580)
48	Evaluating safety, effectiveness, feasibility and cost effectiveness of utilizing digital health technology in traditional medicine provision	0.693 (0.613, 0.759)	0.467 (0.415, 0.513)
49	Studying the comparative effectiveness of different mind-body medicine approaches on mental health	0.693 (0.621, 0.765)	0.457 (0.410, 0.510)
50	Evaluating the prospect of technology integration for monitoring and enhancing traditional medicine interventions (e.g., wearables, telemedicine) to improve deliverability and impact	0.692 (0.598, 0.761)	0.539 (0.512, 0.589)
51	Studying gender-specific patterns in symptom presentation, treatment preferences, and therapeutic outcomes in integrative and traditional medicine	0.692 (0.603, 0.753)	0.539 (0.512, 0.577)
52	Optimising implementation and promotion of systematic reviews and meta-analyses for traditional systems of medicine to make it evidence-based	0.688 (0.601, 0.763)	0.538 (0.512, 0.577)
53	Evaluating the effectiveness of Tai Chi and nutraceutical synergy in addressing sarcopenia	0.688 (0.604, 0.758)	0.436 (0.396, 0.489)
54	Evaluating the effectiveness of mindfulness-based therapies for dementia	0.682 (0.599, 0.755)	0.490 (0.442, 0.532)
55	Assessing safety and effectiveness of self-acupuncture as an intervention for the self-management of chronic conditions	0.681 (0.602, 0.756)	0.407 (0.371, 0.458)
56	Using AI to reliably predict compound interactions with drugs and other herbs resulting in improved safety and efficacy of formulations	0.680 (0.607, 0.757)	0.469 (0.420, 0.515)
57	Identifying the cultural, sociological and gender-related factors that could assist integrating traditional medicine approaches into the health care system	0.680 (0.603, 0.750)	0.466 (0.421, 0.513)
58	Studying the comparative effectiveness of different mind-body therapeutical approaches on chronic pain	0.680 (0.602, 0.747)	0.456 (0.408, 0.500)
59	Evaluating the impact of using herbal medicinal products during pregnancy	0.679 (0.607, 0.754)	0.501 (0.444, 0.540)
60	Studying mind-body therapies for patient-centered outcomes for mental health (e.g., anxiety and depression) to better support whole person health	0.677 (0.599, 0.748)	0.455 (0.412, 0.504)
61	Assessing the impact of a plant-based diet on plasma biomarkers of neurodegenerative diseases	0.676 (0.597, 0.752)	0.447 (0.402, 0.492)

Rank	Research idea	Overall RPS (95%CI)	Overall AEA-e (95%CI)
62	Explore the European Union concepts of "traditional use" and "well-established use" to develop local portfolios of efficient and safe herbs and recipes	0.674 (0.591, 0.752)	0.432 (0.388, 0.483)
63	Studying the effectiveness of holistic and integrative approaches in treating cognitive disorders, such as Alzheimer's disease	0.673 (0.590, 0.737)	0.481 (0.433, 0.523)
64	Studying the comparative effectiveness of different forms of physical activity on chronic pain	0.673 (0.599, 0.743)	0.440 (0.397, 0.481)
65	Evaluating the role of traditional medicine in managing high blood pressure in pregnancy	0.671 (0.592, 0.737)	0.500 (0.451, 0.541)
66	Assessing the safety and dosage of herbal medicine products through clinical trials	0.667 (0.592, 0.739)	0.529 (0.507, 0.565)
67	Systematic reviewing of the data on medicinal and food plants used in traditional medicine that influence quality of life in ageing (e.g., depression, memory loss and weakness).	0.665 (0.588, 0.739)	0.485 (0.436, 0.526)
68	Studying the concept of "being healthy" by different indigenous populations and their traditional and/or other practices to maintain health and treat disease	0.664 (0.588, 0.737)	0.440 (0.397, 0.489)
69	Studying the effectiveness of holistic and integrative approaches in treating neurodegenerative diseases, such as multiple sclerosis	0.660 (0.587, 0.734)	0.447 (0.404, 0.487)
70	Measuring the effects of music-based interventions on brain and overall health and well-being	0.660 (0.572, 0.732)	0.459 (0.412, 0.501)
71	Research on the contribution of traditional health practices and medicinal plants in traumatology	0.659 (0.588, 0.723)	0.404 (0.373, 0.442)
72	Studying the applicability and usefulness of AI in the study of the quality, function, mechanisms and targets of traditional medicine	0.659 (0.574, 0.742)	0.526 (0.505, 0.566)
73	Designing approaches for promoting transparency and collaboration in traditional medicine research through open science initiatives	0.658 (0.581, 0.735)	0.444 (0.399, 0.488)
74	Studying mind-body therapies for patient-centered outcomes for promoting healthy lifestyle and behavior change (e.g., increasing physical activity, dietary change)	0.656 (0.578, 0.727)	0.448 (0.409, 0.489)
75	Studying and mapping availability and geographic distribution of traditional medicine by countries, barriers to services and equity in access	0.655 (0.583, 0.731)	0.444 (0.401, 0.490)
76	Assessing the state of traditional medicine in Africa: Survey of traditional medicine and healthy ageing (setting objectives, conducting reviews and surveys, data collection and analysis, engagement)	0.655 (0.576, 0.735)	0.460 (0.413, 0.505)

Rank	Research idea	Overall RPS (95%CI)	Overall AEA-e (95%CI)
77	Studying the comparative effectiveness of different forms of physical activity on mental health	0.655 (0.581, 0.722)	0.429 (0.387, 0.469)
78	Development of optimal and scalable models of integration of TCI interventions in health care systems	0.652 (0.572, 0.725)	0.439 (0.400, 0.480)
79	Evaluating the complementary role of Ayurvedic therapeutic interventions in the management of specific cancers	0.649 (0.563, 0.720)	0.414 (0.381, 0.462)
80	Standardizing acupuncture methods in treatment of stroke	0.647 (0.568, 0.721)	0.468 (0.420, 0.508)
81	Evaluating the use of antioxidants derived from plants and their byproducts for the prevention of neurodegenerative diseases	0.646 (0.564, 0.714)	0.478 (0.428, 0.523)
82	Health services research and health economic analyses of traditional medicine systems within and outside the respective countries of origin	0.643 (0.555, 0.717)	0.447 (0.402, 0.494)
83	Investigating mechanisms underlying health effects of music (e.g., identifying neural circuitry and biomarkers involved)	0.642 (0.556, 0.733)	0.471 (0.415, 0.514)
84	Evaluating the Impact of traditional medicine as a vital component of global health security	0.639 (0.549, 0.708)	0.520 (0.502, 0.550)
85	Identifying the most appropriate institutional, organizational, and financing options to provide health insurance for traditional medicine services and indemnity for practitioners	0.638 (0.560, 0.716)	0.520 (0.503, 0.547)
86	Evaluating the use of traditional and complementary therapies for assisting smoking cessation	0.638 (0.562, 0.710)	0.435 (0.396, 0.477)
87	Studying the safety and any adverse effects of Yoga practices to improve health and well-being	0.636 (0.561, 0.710)	0.406 (0.373, 0.446)
88	Evaluating the effectiveness of Ayurvedic herbal remedies in improving quality of life and preventing progression of chronic kidney disease	0.635 (0.553, 0.714)	0.474 (0.432, 0.516)
89	Using AI, machine learning and network analyses to understand interactions and the impact of individual genetic profiles on supplement effects (personalized medicine)	0.635 (0.544, 0.710)	0.484 (0.437, 0.525)
90	Comparing the effectiveness, cost-effectiveness, and adherence to integrative and traditional medicine self-care interventions between face-to-face and digital methods	0.635 (0.562, 0.706)	0.501 (0.453, 0.528)
91	Prospective confirmatory clinical studies (randomized controlled trials, cohort studies) on non-pharmacological TCI medicine interventions (e.g., nutrition, lifestyle) for highly prevalent NCDs	0.633 (0.544, 0.718)	0.451 (0.407, 0.491)
92	Conducting a comprehensive and robust health economic evaluation for key forms of TCI interventions	0.630 (0.556, 0.711)	0.446 (0.405, 0.488)

Rank	Research idea	Overall RPS (95%CI)	Overall AEA-e (95%CI)
93	Evaluating the contribution of traditional birth attendants in reducing maternal and neonatal mortality	0.629 (0.550, 0.705)	0.427 (0.391, 0.469)
94	Studying the effectiveness and advantages of traditional Chinese medicine in treating tumors	0.628 (0.547, 0.704)	0.446 (0.406, 0.487)
95	Evaluating the effectiveness of mindfulness-based therapies for improving post-COVID conditions (e.g., long COVID)	0.628 (0.546, 0.716)	0.473 (0.426, 0.509)
96	Studying the user perception of effectiveness of TCI medicine, their effects on life quality, role in recovery and rehabilitation, and effects on long term outcomes)	0.625 (0.536, 0.693)	0.497 (0.448, 0.523)
97	Sustainable development of the products of African traditional medicine against diseases of poverty and ageing (e.g., plants identification, collaboration with healers and communities)	0.620 (0.529, 0.702)	0.515 (0.501, 0.544)
98	Developing and testing mixed methods for evaluating benefits and harms for TCI medicine practices	0.614 (0.536, 0.690)	0.444 (0.401, 0.478)
99	Evaluating the safety and effectiveness of commercially available products in traditional medicine	0.613 (0.535, 0.690)	0.513 (0.501, 0.539)
100	Investigate neuroprotective effects of traditional medicines based on candidates from animal models and known molecular mechanisms	0.606 (0.515, 0.682)	0.478 (0.431, 0.513)
101	Studying innovative use of phytomedicines in the fight against various diseases	0.604 (0.524, 0.670)	0.443 (0.402, 0.476)
102	Development of the policies for protection of the resources for African traditional medicine (with resource assessment, stakeholder engagement, conservation and ethnobotanical studies)	0.602 (0.512, 0.681)	0.490 (0.448, 0.517)
103	Creating a 5-year WHO's global herbal medicine development plan focused on uplifting the lives of farmers and transforming farming communities	0.602 (0.514, 0.682)	0.491 (0.448, 0.519)
104	Assessing the effect of personalization and tailoring of self-care interventions on the outcomes, adherence and costs	0.602 (0.526, 0.688)	0.439 (0.395, 0.475)
105	Designing a population-level study through establishing the national statistical registry for traditional medicine interventions	0.601 (0.519, 0.675)	0.466 (0.417, 0.502)
106	Studying the role of traditional medicine workers in addressing Sustainable Development Goal 3 of "good health and well-being"	0.598 (0.519, 0.677)	0.407 (0.372, 0.439)
107	Evaluating the quality and impact of publications in traditional medicine to move towards informed decision-making	0.597 (0.518, 0.683)	0.510 (0.500, 0.533)

Rank	Research idea	Overall RPS (95%CI)	Overall AEA-e (95%CI)
108	Establishing an optimal global model for integration of traditional medicine into mainstream medical system	0.595 (0.504, 0.674)	0.430 (0.386, 0.468)
109	Exploring the optimal supply chains and delivery strategies of traditional herbal medicines by comparing local cultivation versus importing/exporting	0.593 (0.504, 0.674)	0.450 (0.403, 0.487)
110	Developing criteria for selection of the most promising plant species for effectiveness evaluation in clinical studies	0.589 (0.508, 0.669)	0.508 (0.500, 0.530)
111	Scientific searching for innovative herbal preparations with antiviral activity against the SARS-CoV-2 coronavirus for complex treatment and prevention	0.585 (0.496, 0.671)	0.462 (0.421, 0.500)
112	Exploring how can indirect risks and contextual factors associated with TCI medicine be addressed in regulatory and policy frameworks to minimize these risks	0.585 (0.500, 0.669)	0.507 (0.500, 0.527)
113	Studying how gender-specific patterns inform personalized and gender-tailored interventions in integrative and traditional medicine	0.575 (0.500, 0.654)	0.445 (0.405, 0.478)
114	Investigating and documenting the most frequent adverse effects of TCI medicine, patient satisfaction and adherence rate, and reduction of side effects of conventional drugs	0.575 (0.480, 0.651)	0.488 (0.445, 0.511)
115	Evaluating the use of traditional foods as a medicine for long-term care for major non-communicable diseases	0.573 (0.485, 0.653)	0.428 (0.389, 0.463)
116	Evaluating the effect of demographic variables on the efficacy of integrative and traditional medicine self-care digital interventions	0.572 (0.496, 0.656)	0.436 (0.395, 0.468)
117	Developing guidelines to increase public awareness about extending healthy longevity of people, taking into account innovative traditional medicine approaches	0.566 (0.485, 0.647)	0.504 (0.500, 0.522)
118	Encourage researchers from all countries to conduct in-depth ethnobotanical studies on medicinal plants used by women, both to compile information and safeguard the traditional knowledge on remedies applied to their specific health problems.	0.564 (0.485, 0.652)	0.485 (0.439, 0.508)
119	Studying the knowledge, acceptance, and use of traditional medicine among health care professionals, decision makers, patients and general population	0.562 (0.476, 0.637)	0.437 (0.399, 0.468)

Rank	Research idea	Overall RPS (95%CI)	Overall AEA-e (95%CI)
120	Assessing changes in plasma Neuropeptide Y and Cortisol following both acute and chronic acupuncture treatments	0.562 (0.488, 0.637)	0.365 (0.344, 0.392)
121	Evaluating the application of health technology assessment for traditional medicine and traditional health practices	0.561 (0.482, 0.636)	0.415 (0.379, 0.451)
122	Evaluating the potential impact of TCI medicine on overall disease burden, for improving heath indicators, influencing medications use, and affecting healthcare costs	0.560 (0.472, 0.630)	0.443 (0.398, 0.474)
123	Studying the effects of traditional medicine in the treatment of rare and difficult-to-treat diseases	0.554 (0.463, 0.630)	0.485 (0.447, 0.506)
124	Evaluating therapeutic effects and action mechanisms of polysaccharides in traditional medicines	0.554 (0.459, 0.636)	0.468 (0.422, 0.503)
125	Studying the effects of polysaccharides from traditional medicines on nervous diseases such as epilepsy	0.548 (0.456, 0.631)	0.468 (0.423, 0.501)
126	Studying the demand at the national and local level for TCI medicine (including acceptable frequency of use, socioeconomic and ethnicity influences)	0.545 (0.460, 0.626)	0.434 (0.394, 0.470)
127	Mapping the key components of the TCI medicine therapeutic encounters and using this knowledge to integrate them into the conventional healthcare consultation	0.543 (0.461, 0.618)	0.408 (0.376, 0.440)
128	Studying the comparative effectiveness of different forms of diet on mental health	0.542 (0.458, 0.626)	0.447 (0.404, 0.481)
129	Developing and testing hydrophilic natural neuroprotective compounds for targeted penetration of blood-brain barrier	0.541 (0.463, 0.632)	0.435 (0.394, 0.470)
130	Studies to identify optimal ways for protecting intellectual properties of communities and indigenous peoples	0.540 (0.456, 0.624)	0.470 (0.430, 0.501)
131	Studying the effects of traditional medicine in promoting healthy longevity.	0.539 (0.461, 0.618)	0.502 (0.500, 0.514)
132	Evaluating the effectiveness of millet flours fortified with relevant medicinal herbs on plasma markers of metabolic disorders	0.538 (0.453, 0.620)	0.425 (0.388, 0.464)
133	Evaluating the effectiveness of traditional ceremonial practices as a strategy to reduce substance abuse in American Indian Communities	0.538 (0.455, 0.622)	0.447 (0.403, 0.481)
134	Evaluating the usefulness of Health Resort medicine's impact on healthy ageing	0.525 (0.439, 0.615)	0.426 (0.385, 0.462)
135	Identifying the most critical behavioural insights and nudges related to traditional medicine use	0.523 (0.439, 0.610)	0.501 (0.500, 0.510)

Rank	Research idea	Overall RPS (95%CI)	Overall AEA-e (95%CI)
136	Evaluating the effectiveness of distance healing (e.g., Reiki) for reducing refugee anxiety	0.521 (0.432, 0.614)	0.432 (0.389, 0.465)
137	Developing antivirulence strategies from the anti-infectious cocktails produced by herbal drugs	0.519 (0.440, 0.612)	0.481 (0.427, 0.501)
138	Developing and testing novel formulations for natural products, including nanoparticles, to increase their absorption and distribution in mammals	0.511 (0.422, 0.593)	0.422 (0.381, 0.450)
139	Exploratory and applied research of external therapy in traditional Chinese medicine	0.508 (0.419, 0.593)	0.442 (0.397, 0.472)
140	Evaluating integrative pharmacological effects of multiple components in Traditional medicine	0.508 (0.432, 0.601)	0.481 (0.429, 0.501)
141	Studying the integrative pharmacological effects of multiple components in traditional medicines	0.504 (0.419, 0.599)	0.480 (0.428, 0.501)
142	Evaluating the impact of Health Resort interventions and Ginko biloba nutraceutical supplementation on sarcopenia and frailty in older individuals	0.500 (0.414, 0.584)	0.425 (0.384, 0.454)
143	Developing guidelines for quality control of polysaccharides in traditional medicine	0.500 (0.410, 0.598)	0.500 (0.500, 0.501)
144	Examining traditional medical providers' perceptions and experience with medical misinformation in scientific literature and the impact on clinical decisions	0.500 (0.429, 0.581)	0.409 (0.376, 0.435)
145	Improving methods for improving gender equity and inclusivity in integrative medicine research to generalise its application	0.496 (0.410, 0.575)	0.481 (0.439, 0.501)
146	Collection and analysis of retrospective and prospective data that may identify interventions that improve health trajectories for particular conditions	0.496 (0.409, 0.575)	0.401 (0.367, 0.435)
147	Exploring the modulation of DNA damage translesion synthesis and repair by natural products	0.492 (0.408, 0.584)	0.416 (0.375, 0.443)
148	Evaluating the role of traditional medicine in managing excessive blood loss in pregnancy	0.483 (0.402, 0.565)	0.433 (0.394, 0.460)
149	Investigating the influence of advertised traditional medicine products on the use of existing medications or delaying early medical intervention	0.479 (0.401, 0.563)	0.500 (0.500, 0.502)
150	Mapping changing needs for TCI over a lifetime and related gender differences	0.467 (0.380, 0.554)	0.434 (0.393, 0.468)
151	Systematic exploration of data on chemically similar plant species that occur throughout the world and that could also be evaluated	0.460 (0.375, 0.548)	0.467 (0.419, 0.501)
152	Exploring the food and beverage intake history of a large multinational cohort of patients with dementia and Alzheimer's disease	0.456 (0.376, 0.540)	0.440 (0.400, 0.472)

Rank	Research idea	Overall RPS (95%CI)	Overall AEA-e (95%CI)
153	Investigating the impact of advertised Traditional medicine products on self-treatment in the population	0.448 (0.376, 0.526)	0.400 (0.369, 0.431)
154	Studying how is interculturality perceived and practiced between conventional and traditional medicine providers	0.434 (0.352, 0.525)	0.504 (0.500, 0.523)
155	Agreeing on formulating contextualised research questions (e.g., "exercise" in high-income vs. manual labor to account for class, socio-economic and cultural differences)	0.433 (0.345, 0.517)	0.437 (0.393, 0.472)
156	Evaluating the use of natural products or byproducts for the enrichment of animal feed	0.422 (0.333, 0.504)	0.506 (0.500, 0.526)
157	Studying and classifying by their quality any claims and product categories that are being advertised in press and on the internet	0.408 (0.324, 0.489)	0.477 (0.433, 0.510)

Note: *RPS, research priority score; AEA-e, average expert agreement score; CI, confidence interval; TCI, traditional, complementary and integrative; NCDs, non-communicable diseases; WHO, World Health Organization; AI, artificial intelligence.*

Table S4. The output from the three LLMs for TCI medicine research priorities

Prompt: *Please define and list, in order of importance, the top 10 research priorities for traditional, complementary, and integrative (TCI) medicine worldwide.*

ChatGPT 4o

Defining global research priorities for Traditional, Complementary, and Integrative (TCI) medicine involves considering the public health relevance, scientific feasibility, potential for global impact, and integration with conventional healthcare systems. The World Health Organization (WHO) and various research organizations have identified broad goals in this domain. Based on global trends, policy directions, and emerging needs, the following is a proposed list of the **top 10 research priorities** for TCI medicine worldwide, **in order of importance**:

1. Evidence Generation for Safety and Efficacy

• **Why it's top priority**: Scientific validation of TCI practices is essential for public trust, clinical use, and regulatory integration.
• **Focus**: High-quality clinical trials, systematic reviews, and real-world evidence on the efficacy and safety of TCI interventions.

2. Integration of TCI into Primary Health Care Systems

• **Why**: Integration can improve healthcare access, patient satisfaction, and holistic outcomes.
• **Focus**: Implementation science, health systems research, models for integrated care delivery, and cost-effectiveness analyses.

3. Regulation, Quality Assurance, and Standardisation

• **Why**: Ensures safety, consistency, and trust in TCI products and practices.
• **Focus**: Pharmacopoeias, standards for herbal medicines, practitioner accreditation, and regulation of traditional products.

4. Mechanisms of Action and Translational Research

- **Why**: Understanding biological mechanisms can bridge TCI and biomedicine.
- **Focus**: Molecular biology, systems biology, and omics approaches to investigate how TCI interventions work.

5. Addressing Non-Communicable Diseases (NCDs)

- **Why**: NCDs are the leading cause of global mortality, and TCI can contribute to prevention and management.
- **Focus**: Role of TCI in cardiovascular diseases, diabetes, mental health, chronic pain, and cancer care.

6. TCI in Mental Health and Wellbeing

- **Why**: Rising burden of mental health disorders and growing use of mind-body therapies.
- **Focus**: Mindfulness, meditation, acupuncture, yoga, and herbal treatments in anxiety, depression, stress, and neurodegenerative conditions.

7. Indigenous Knowledge Systems and Biodiversity Conservation

- **Why**: Preserves cultural heritage and biodiversity while supporting sustainable healthcare.
- **Focus**: Documentation of indigenous practices, ethical research collaborations, and intellectual property rights.

8. Personalized and Preventive Health Approaches

- **Why**: TCI often emphasizes individualized care and prevention.
- **Focus**: Ayurvedic or traditional diagnostic systems, lifestyle interventions, and preventive care models.

9. Digital Health and Innovation in TCI

• **Why**: Technology can enhance access, education, and data generation.
• **Focus**: AI in herbal medicine discovery, digital diagnostics, mobile health apps for TCI, and virtual training for practitioners.

10. Health Equity, Access, and Cultural Acceptability

• **Why**: Ensures inclusive, respectful care that reaches underserved populations.
• **Focus**: Role of TCI in rural and marginalized communities, sociocultural research, and patient-centered outcomes.

Claude 3.5

Top 10 Research Priorities for Traditional, Complementary, and Integrative (TCI) Medicine

1. Efficacy and Safety Evaluation

The most critical research priority is conducting rigorous, high-quality scientific studies to:

- Systematically evaluate the efficacy and safety of traditional and complementary medical interventions
- Develop standardized methodologies for assessing complex interventions
- Create robust evidence-based frameworks for understanding TCI treatments
- Identify potential interactions with conventional medical treatments
- Establish clear safety protocols and potential contraindications

2. Mechanistic Understanding

Investigating the underlying biological mechanisms of TCI interventions is crucial for:

- Elucidating how traditional therapies produce physiological effects
- Understanding molecular and cellular pathways triggered by complementary treatments
- Bridging the gap between traditional knowledge and modern scientific understanding
- Developing potential new therapeutic approaches based on discovered mechanisms
- Creating more precise and targeted integrative treatment strategies

3. Global Health Integration

Developing comprehensive strategies for integrating TCI into mainstream healthcare systems worldwide, including:

- Creating policy frameworks for TCI integration
- Establishing standardized educational and training programs
- Developing interdisciplinary collaboration models
- Addressing cultural and systemic barriers to integration
- Ensuring equitable access to integrative healthcare approaches

4. Patient-Centered Outcomes Research

Focusing on patient experiences, quality of life, and holistic health outcomes by:

- Conducting comprehensive patient-reported outcome studies
- Exploring patient perceptions and experiences with TCI treatments
- Developing patient-centered evaluation metrics

- Understanding psychological and social dimensions of healing
- Investigating long-term health impacts of integrative approaches

5. Pharmacological and Botanical Research

Advancing scientific understanding of traditional medicinal substances through:

- Phytochemical analysis of medicinal plants
- Identifying active compounds and their therapeutic potential
- Developing standardized extraction and preparation methods
- Exploring potential new drug discoveries
- Investigating interactions between botanical compounds and conventional medications

6. Precision and Personalized Medicine

Developing tailored integrative approaches that consider individual variability by:

- Investigating genetic and metabolic factors influencing treatment responses
- Creating personalized treatment algorithms
- Developing predictive models for treatment effectiveness
- Exploring individualized nutrition and lifestyle interventions
- Understanding complex interactions between genomics and traditional healing practices

7. Mental Health and Psychological Interventions

Examining TCI approaches for mental health and psychological well-being, including:

- Studying mindfulness and meditation practices

- Investigating traditional mind-body interventions
- Exploring holistic approaches to mental health treatment
- Developing integrative mental health protocols
- Understanding neuroplasticity and psychological healing mechanisms

8. Chronic Disease Management

Researching TCI strategies for managing chronic and complex health conditions:

- Developing integrative approaches for diabetes, cardiovascular diseases, and cancer
- Investigating holistic lifestyle interventions
- Creating comprehensive management strategies
- Exploring prevention and supportive care approaches
- Understanding the role of traditional practices in long-term health management

9. Global Traditional Knowledge Preservation

Documenting and preserving traditional medical knowledge through:

- Comprehensive ethnographic research
- Creating digital archives of traditional medical practices
- Supporting indigenous knowledge systems
- Developing ethical frameworks for knowledge documentation
- Protecting intellectual property rights of traditional medical communities

10. Economic and Health System Impact

Analyzing the broader implications of TCI integration:

- Conducting health economic evaluations
- Investigating cost-effectiveness of integrative approaches
- Developing health policy recommendations
- Exploring workforce development and training
- Understanding potential healthcare system transformations

Grok 3

Top 10 Research Priorities for Traditional, Complementary, and Integrative (TCI) Medicine

1. Developing Standardized Research Methodologies and Protocols for TCI Medicine

A major barrier in TCI medicine is the lack of consistent research methods. Creating standardized protocols would ensure studies are rigorous, reproducible, and comparable, providing a solid foundation for credible evidence.

2. Improving Research Capacity and Training for TCI Medicine Practitioners and Researchers

Limited training in research skills hinders progress in the field. Enhancing educational programs and capacity-building initiatives would equip practitioners and researchers to conduct high-quality studies, advancing TCI medicine's credibility.

3. Advocating for Increased Funding and Resource Allocation for TCI Medicine Research

Financial constraints restrict the scope and scale of TCI research. Securing more funding would enable larger, impactful studies to address critical questions and expand the evidence base.

4. Conducting Research on the Effectiveness and Safety of TCI Medicine Interventions

Proving the safety and efficacy of TCI practices is essential for gaining acceptance among healthcare professionals and integrating these approaches into mainstream care. This research would counter biases and build trust.

5. Researching the Efficacy of TCI Medicine for Specific Health Conditions

Evidence is needed to show how TCI medicine can treat or manage specific conditions, such as chronic diseases, mental health issues, or respiratory illnesses. This would support its targeted use in clinical settings.

6. Researching Policy Development and Implementation for the Integration of TCI Medicine into National Health Systems

Integrating TCI medicine into healthcare systems requires evidence-based policies. Research in this area would guide the creation of frameworks to ensure safe and effective adoption on a systemic level.

7. Researching Patient-Centered Care and Individualized Approaches in TCI Medicine

TCI medicine's holistic focus emphasizes personalized care. Studying patient engagement and tailored treatments would ensure these practices meet individual needs and improve outcomes.

8. Exploring the Potential of TCI Medicine in Preventive Care and Health Promotion

With its emphasis on well-being and prevention, TCI medicine could reduce chronic disease burdens. Research here would highlight its value in lifestyle management and proactive health strategies.

9. Investigating the Role of TCI Medicine in Addressing Health Disparities and Improving Access to Care

TCI medicine could improve healthcare equity, especially in underserved areas. Research on its accessibility and impact in diverse populations would promote inclusivity in healthcare delivery.

10. Researching the Integration of TCI Medicine with Conventional Medicine to Create a More Holistic and Comprehensive Healthcare System

Combining TCI and conventional approaches could enhance patient-centered care. Research on integration models would support the development of a more comprehensive healthcare system.

Note: *TCI, traditional, complementary and integrative; LLMs, large language models; NCDs, non-communicable diseases; WHO, World Health Organization; AI, artificial intelligence.*

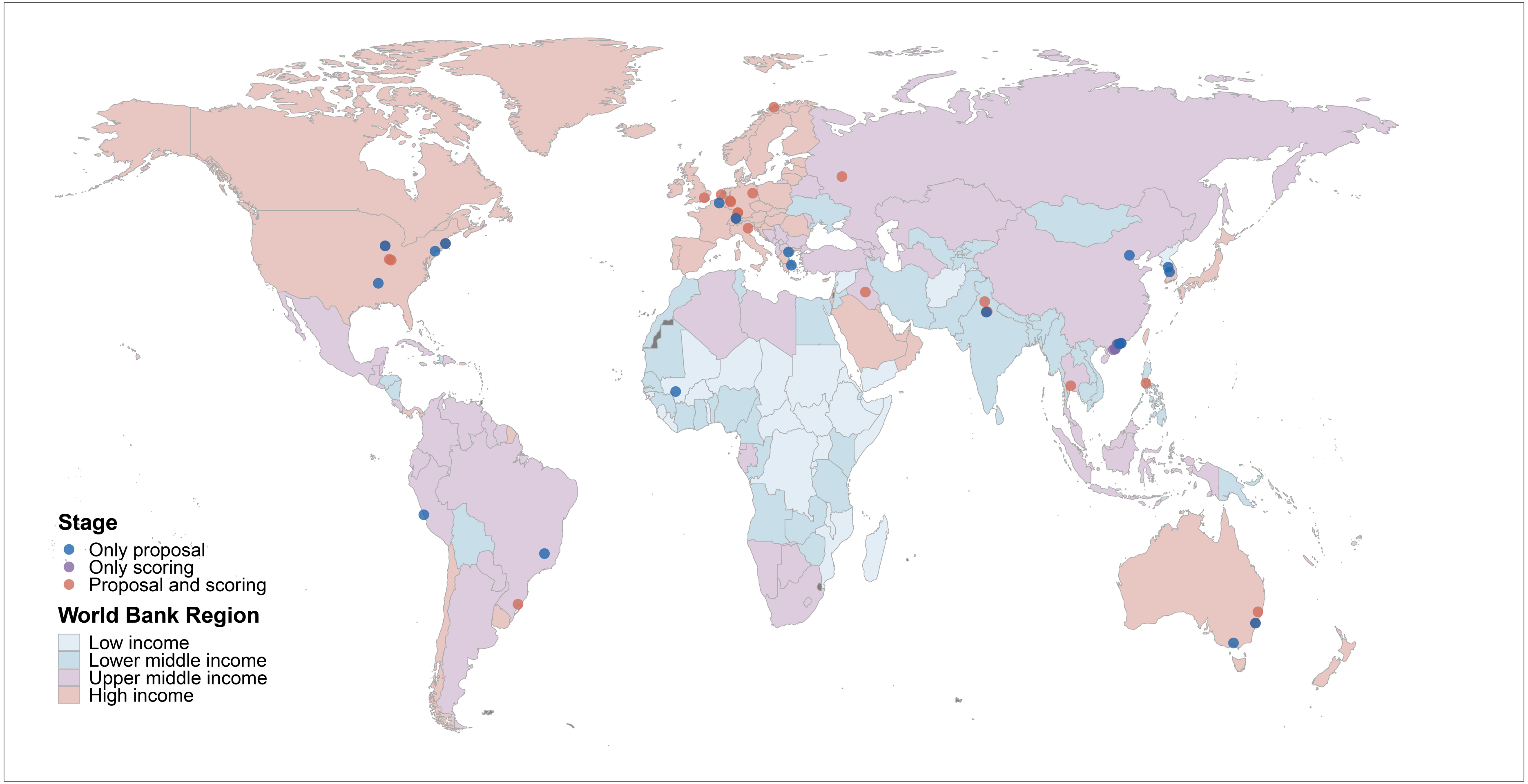

Figure S1. The geographic distribution of contributing experts (N = 53)

Note: *In the research idea generation phase, 44 experts submitted research ideas as requested, and 34 experts participated in the scoring phase.*

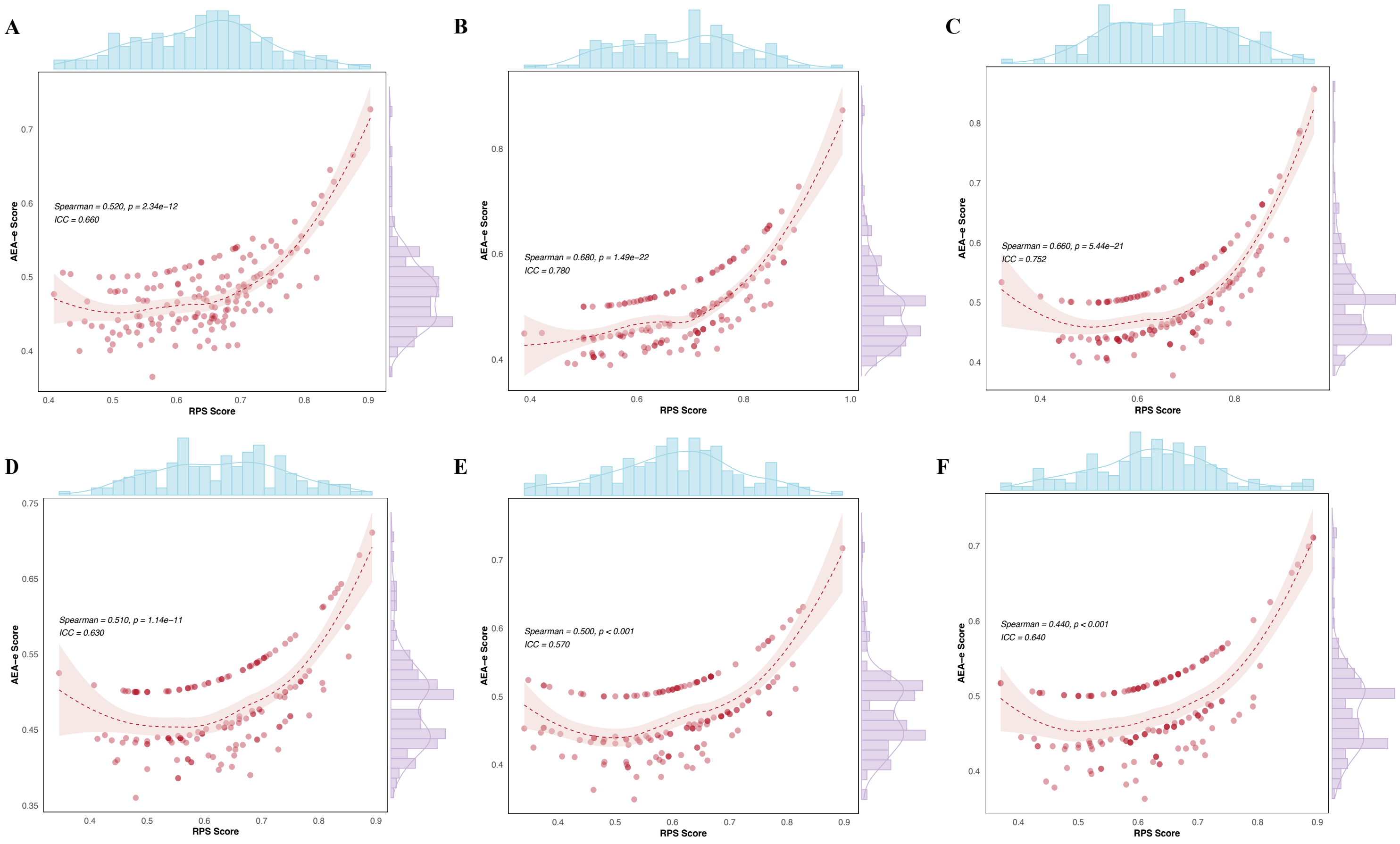

Figure S2. Correlation between RPS and AEA-e

Note: *RPS, research priority score; AEA-e, average expert agreement score; ICC, intraclass correlation coefficient.* ***Panel A****, overall;* ***Panel B****, feasibility and answerability;* ***Panel C****, efficacy and effectiveness;* ***Panel D****, deliverability, affordability, and sustainability;* ***Panel E****, maximum impact on disease burden reduction;* ***Panel F****, impact on equity.*

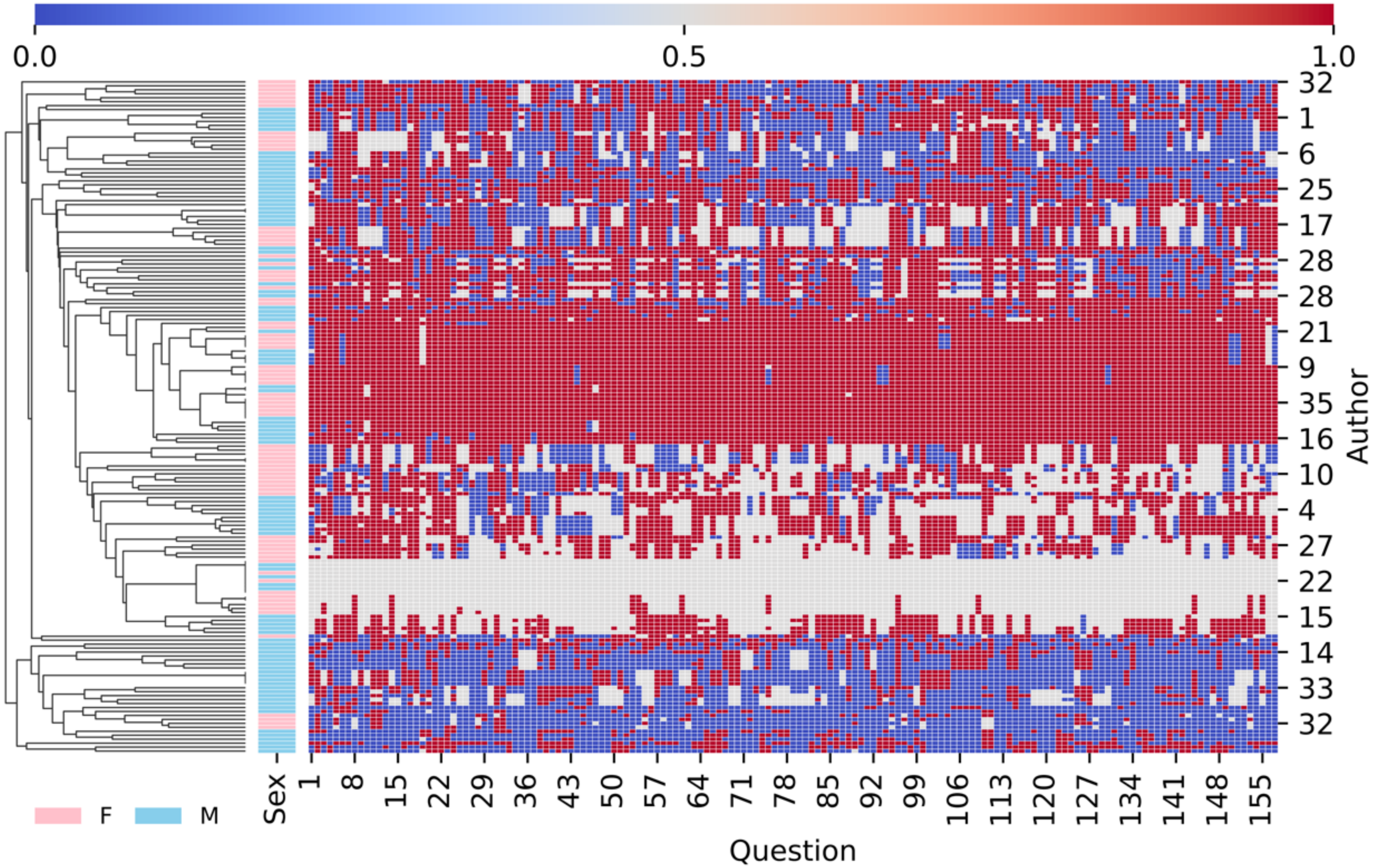

Figure S3. The hierarchical clustering of all idea scores from contributing experts

Note: *F, female; M, male.*

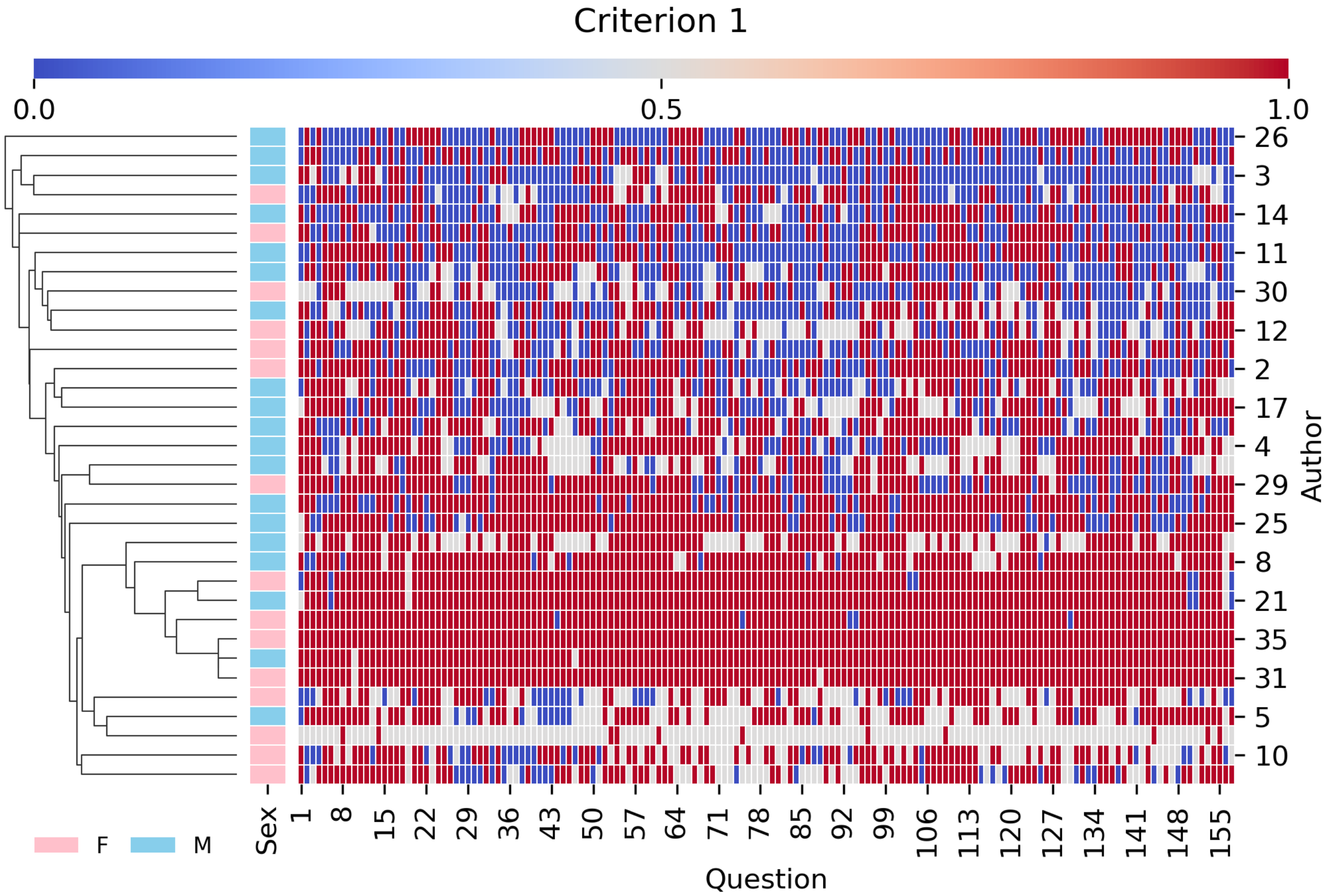

Figure S4. The clustering of idea scores in the Criterion 1 – Feasibility and Answerability

Note: *F, female; M, male.*

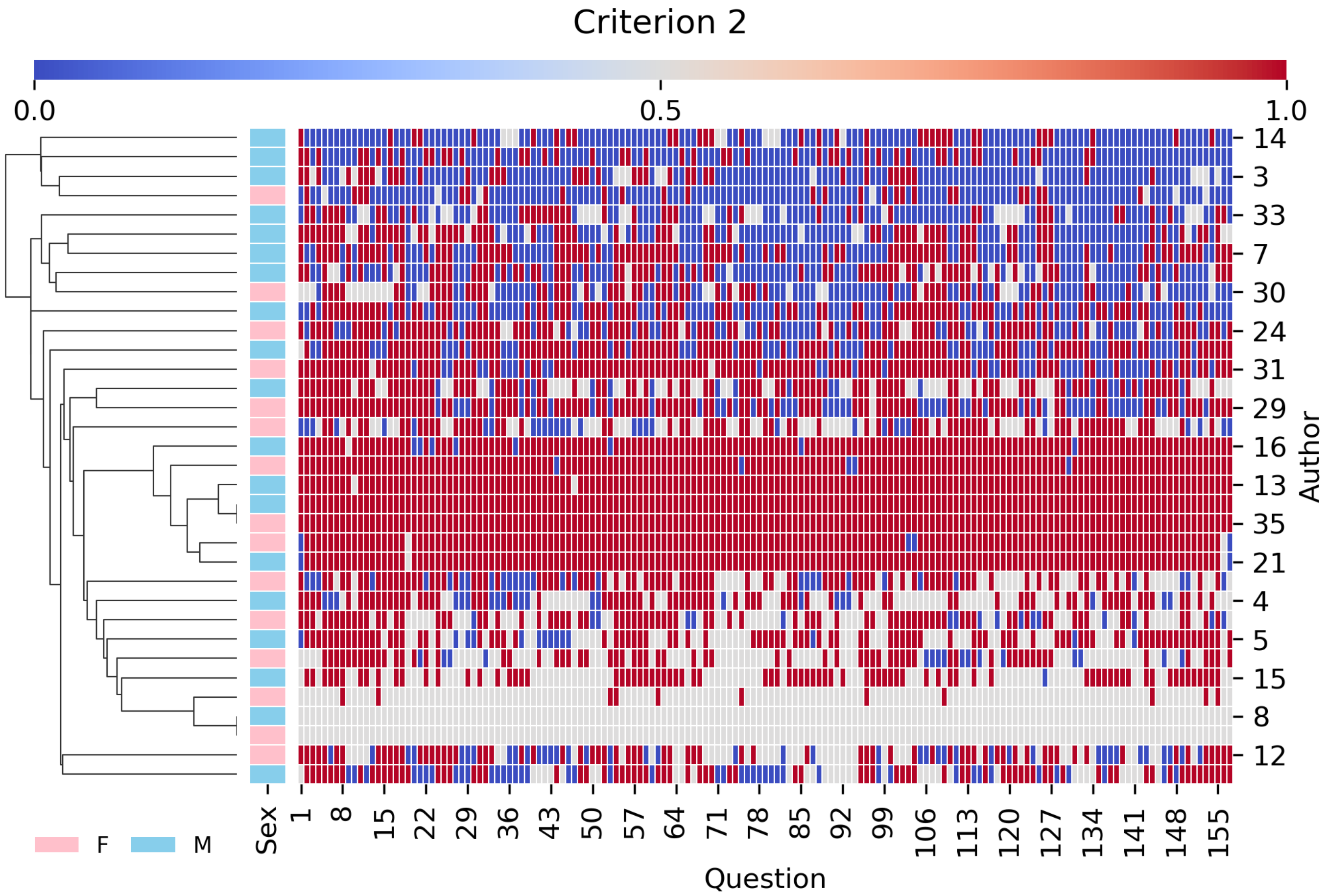

Figure S5. The clustering of idea scores in the Criterion 2 – Efficacy and Effectiveness

Note: *F, female; M, male.*

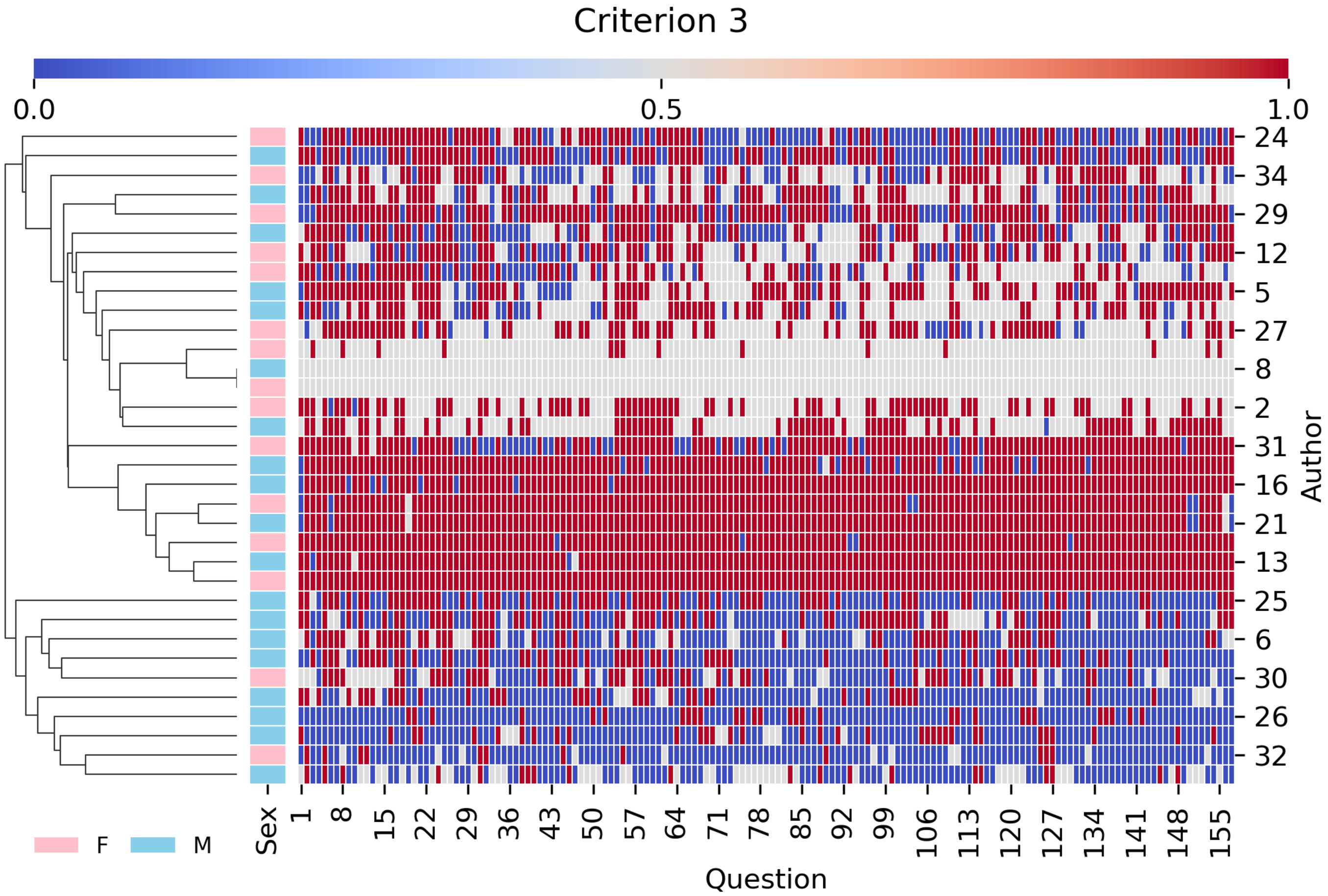

Figure S6. The clustering of idea scores in the Criterion 3 – Deliverability, Affordability, and Sustainability

Note: *F, female; M, male.*

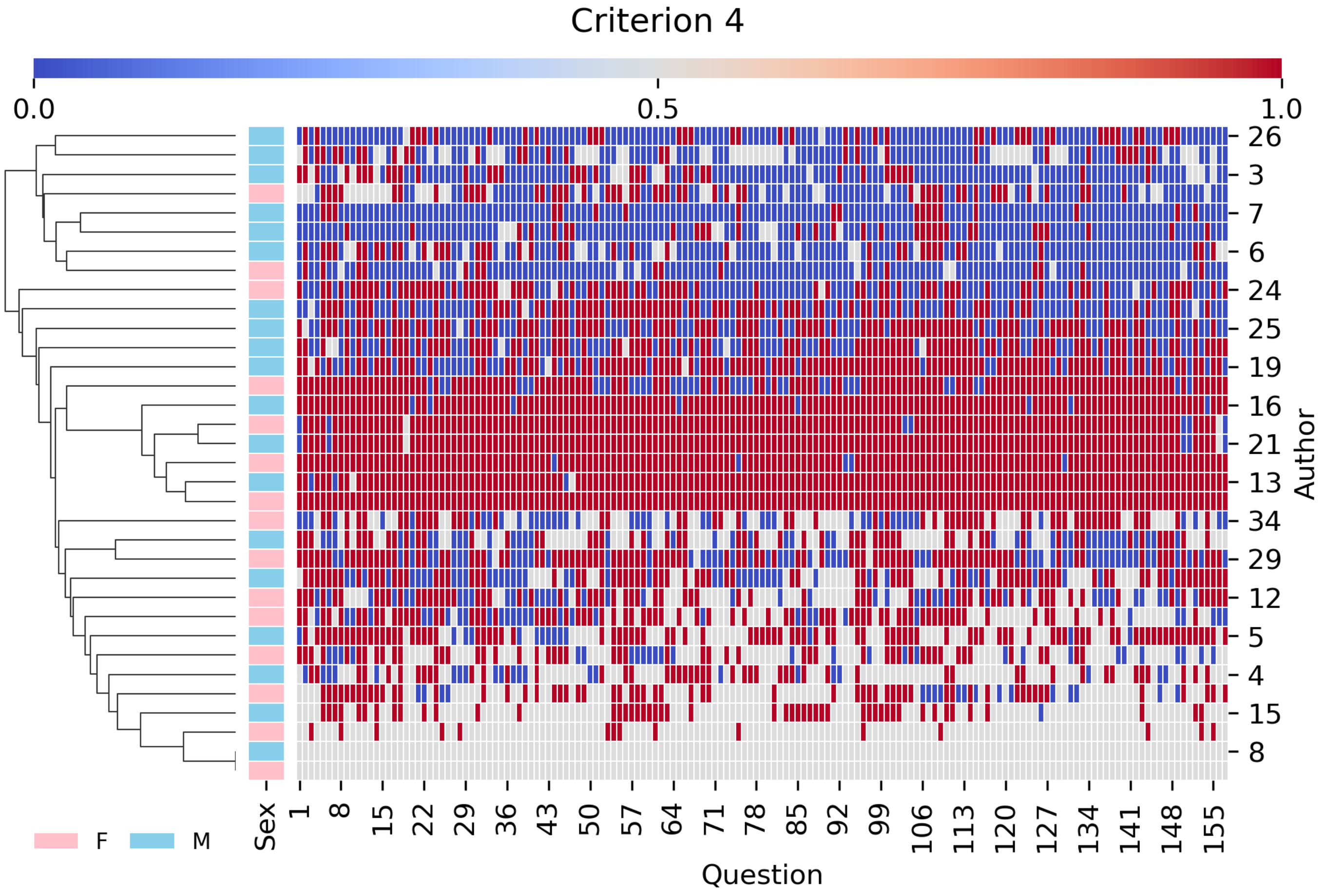

Figure S7. The clustering of idea scores in the Criterion 4 – Maximum Impact on Disease Burden Reduction

Note: *F, female; M, male.*

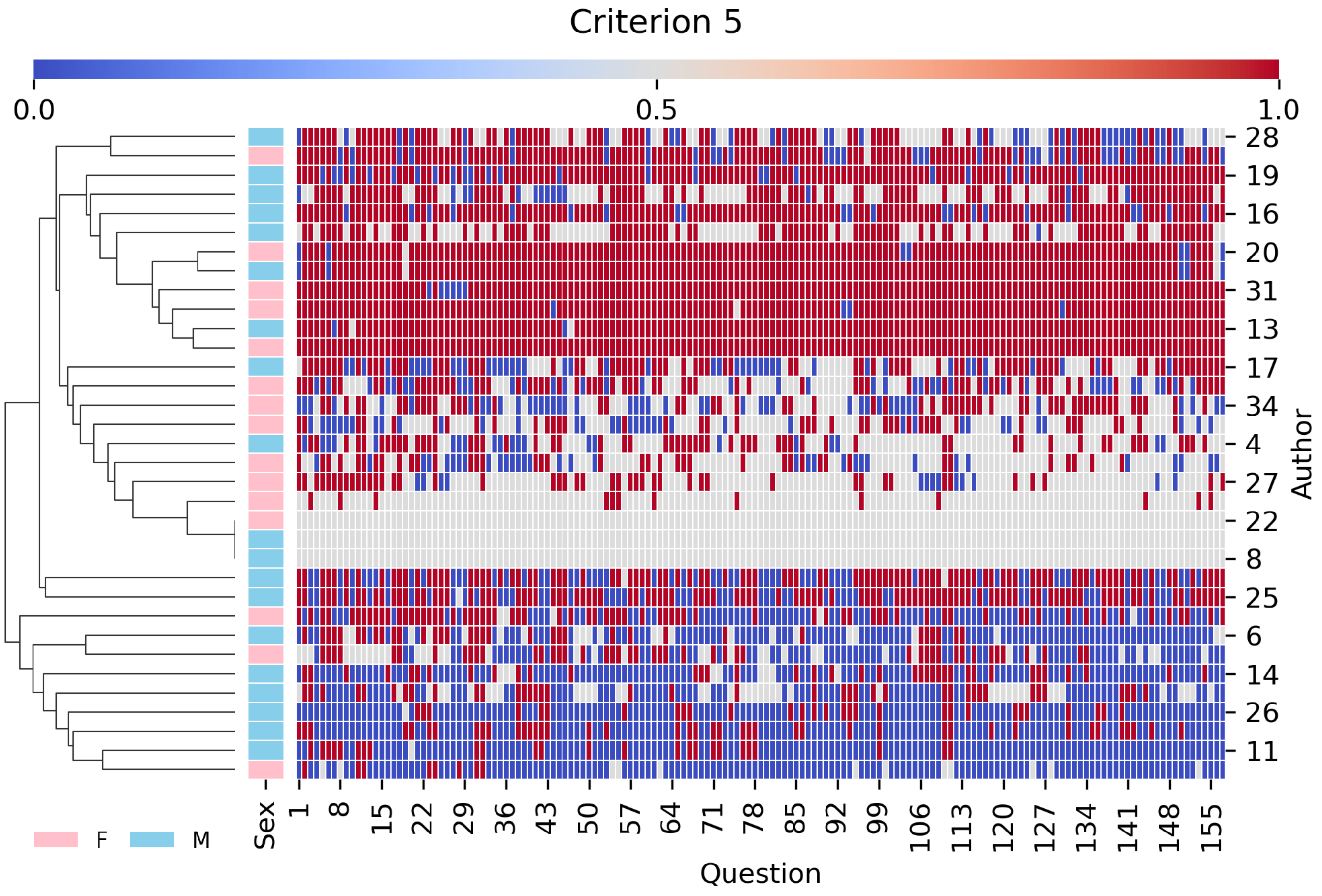

Figure S8. The clustering of idea scores in the Criterion 5 – Impact on Equity

Note: *F, female; M, male.*
